# GV-971 remodels the gut microbiota-bile acid-FXR axis to ameliorate obesity and metabolic dysfunction

**DOI:** 10.1038/s41421-026-00904-6

**Published:** 2026-07-21

**Authors:** Na Zhang, Xinyu Ye, Kai Wang, Yameng Hu, Zixi Wang, Lichun Huang, Xi Chen, Ding Yan, Wen Fu, Qian Xue, Shihao Sun, Yihan Xu, Daolin Tang, Xin Chen, Li Zhou, Jinbao Liu

**Affiliations:** 1 Guangzhou Municipal and Guangdong Provincial Key Laboratory of Protein Modification and Disease, State Key Laboratory of Respiratory Disease, School of Basic Medical Sciences, Guangzhou Medical University, Guangdong, Guangzhou China; 2 Guangdong Provincial Key Laboratory of Protein Modification and Disease, Key Laboratory of Biological Targeting Diagnosis, Therapy and Rehabilitation of Guangdong Higher Education Institutes, School of Basic Medical Sciences and The Fifth Affiliated Hospital, Guangzhou Medical University, Guangdong, Guangzhou China; 3 Department of Surgery, UT Southwestern Medical Center, Dallas, TX USA

**Keywords:** Mechanisms of disease, Transcription

## Abstract

Obesity and its associated metabolic complications represent a global health crisis, yet effective microbiota-targeted pharmacotherapies remain limited. Here, we report that GV-971 (sodium oligomannate), a marine-derived oligosaccharide originally developed for Alzheimer’s disease, exerts potent anti-obesity and metabolic benefits by reprogramming gut microbial and host signaling networks. In high-fat diet-induced obese mice, GV-971 reduced adiposity, improved glucose homeostasis, and alleviated hepatic steatosis without affecting food intake. Multi-omics and causal intervention experiments revealed that GV-971 selectively decreased the abundance of *Clostridium scindens*, a keystone bacterium responsible for secondary bile acid synthesis. This decrease downregulated the expression of the *baiF* gene encoding 7α-hydroxysteroid dehydrogenase, leading to reduced intestinal deoxycholic acid (DCA) levels and inhibition of intestinal farnesoid X receptor (FXR) signaling. Restoration of *C. scindens* abundance, *baiF* expression, or DCA supplementation abrogated the metabolic benefits of GV-971, confirming the causal role of the *C. scindens*-DCA-FXR axis. Mechanistically, inhibition of intestinal FXR promoted thermogenic gene expression and white adipose tissue browning, thus enhancing systemic energy expenditure. These findings uncover a bacterium-metabolite-host signaling pathway underlying the effects of GV-971 and establish microbiota-directed FXR modulation as a promising therapeutic approach for obesity and metabolic disease.

## Introduction

Obesity and its associated metabolic syndromes, including insulin resistance and metabolic dysfunction-associated steatotic liver disease, represent a major public health burden with limited therapeutic options^1–4^. These disorders arise from complex interactions between nutrient excess, chronic low-grade inflammation, and disrupted energy metabolism^5,6^. Recent advances have revealed that the gut microbiota is a key regulator of host metabolic homeostasis, acting as a metabolic and immunologic interface between diet and peripheral tissues^7–9^. A high-fat diet (HFD) induces gut dysbiosis, increases intestinal permeability, and alters microbial metabolites such as bile acids and short-chain fatty acids (SCFAs), thus propagating systemic inflammation and metabolic dysfunction^10–12^. Consequently, targeting the gut microbiota to restore metabolic balance has emerged as a promising strategy to treat obesity and its complications^13–16^.

Although interventions such as probiotics, prebiotics, and fecal microbiota transplantation (FMT) can partially improve metabolic phenotypes, pharmacological approaches capable of selectively remodeling the gut microbial composition and function remain limited^17,18^. This translational gap underscores the need to identify specific bacterial taxa and metabolite-driven signaling pathways that causally link microbiota modulation to metabolic benefits.

GV-971 (sodium oligomannate), a marine-derived oligosaccharide isolated from brown algae, was initially granted conditional approval in China for the treatment of mild-to-moderate Alzheimer’s disease^19^. Mechanistically, GV-971 is proposed to remodel the gut microbiota, thus suppressing peripheral Th1 cell-mediated inflammation and attenuating neuroinflammation in the central nervous system^20^. Subsequent studies have indicated that GV-971 may also confer protection against acute pancreatic injury through gut-immune signaling pathways^21^. Nevertheless, whether GV-971 can similarly modulate gut–liver–adipose communication to ameliorate obesity-associated metabolic dysfunction remains unclear.

Here, we identify a distinct bacterium-metabolite-signal axis through which GV-971 ameliorates obesity and metabolic dysfunction in diet-induced obese (DIO) mice. GV-971 selectively suppresses the abundance of *Clostridium scindens*, a key bacterium involved in secondary bile acid synthesis. This effect leads to downregulation of the bacterial *baiF* gene encoding 7α-hydroxysteroid dehydrogenase, resulting in reduced intestinal levels of deoxycholic acid (DCA) and subsequent inhibition of intestinal farnesoid X receptor (FXR) activation. Suppression of intestinal FXR signaling decreases ceramide synthesis, thus promoting the expression of thermogenic genes, such as uncoupling protein 1 (*Ucp1*), and inducing beige adipocyte formation within white adipose tissue.

## Results

### GV-971 reduces obesity and metabolic dysfunction in DIO mice

**Fig. 1:**
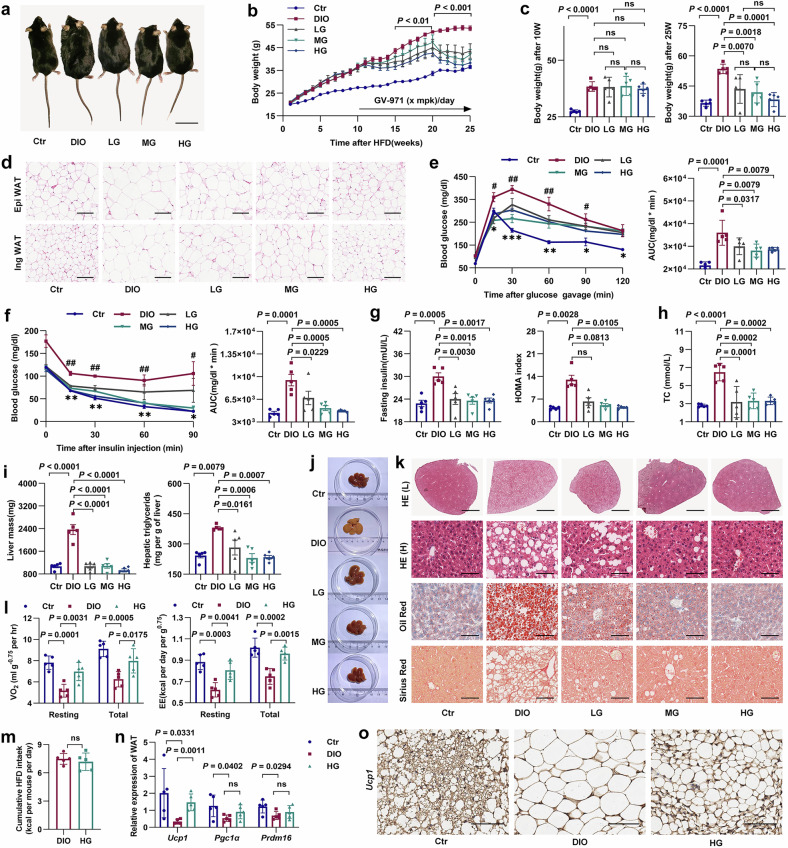
GV-971 effectively ameliorates obesity and metabolic dysfunction in DIO mice. **a** Representative mice in different groups, including control mice treated with vehicle (Ctr, *n* = 5), HFD-induced obese mice (DIO mice) treated with vehicle (DIO, *n* = 5), DIO mice treated with GV-971 (100 mg/kg) (LG, *n* = 5), DIO mice treated with GV-971 (200 mg/kg) (MG, *n* = 5), and DIO mice treated with GV-971 (400 mg/kg) (HG, *n* = 5). Scale bar, 5 mm. **b** Growth curves of body weight. **c** Body weight difference statistical chart of mice fed an HFD after 10 weeks (left) and body weight difference statistical chart of established obese mice fed an HFD treated with vehicle and GV-971 for 15 weeks (right, HFD after 25 weeks). **d** Representative hematoxylin and eosin (H&E) staining of epididymis white adipose tissue (WAT) sections (upper panel, Epi) and inguinal WAT sections (lower panel, Ing). Scale bars, 100 μm. **e** Glucose tolerance test (left) and the area under the curve (AUC) (right). **f** Insulin tolerance test results and the AUC (right). **g** Fasting serum insulin levels (left) and the HOMA-IR test (right). **h** Serum total cholesterol (TC) level. **i** Liver weights (left) and liver triglyceride contents (right). **j** Representative livers from the different groups. **k** Representative H&E staining, oil red staining, and Sirius red staining of liver sections. HE (L) scale bars, 2000 μm; other scale bars, 100 μm. **l** Resting and total O_2_ consumption (left, VO_2_) and resting and total energy expenditure (right, EE). **m** Cumulative HFD intake. **n** mRNA expression of thermogenic genes in inguinal WAT. **o** Representative Ucp1 immunohistochemical staining of inguinal WAT sections from mice. Scale bars, 50 μm.

To determine whether GV-971 could ameliorate HFD-induced obesity, insulin resistance, and hepatic steatosis, DIO mice were orally administered low, medium, or high doses of GV-971 for 15 weeks. Compared with vehicle treatment, GV-971 treatment attenuated body weight gain in DIO mice (Fig. 1a–c) and reduced total fat mass and adipocyte size (Fig. 1d; Supplementary Fig. S1a). Notably, although direct comparisons between the high-dose group (HG) and the low/medium-dose group (LG/MG) did not reach statistical significance, the linear mixed effects model revealed that its weight-reducing effect progressively strengthened over time relative to that of the lower-dose group. Moreover, the high dose had already entered the plateau phase, reaching ~94% of the maximum effect value, indicating that further dose escalation would provide only very limited marginal benefit (Supplementary Tables S1–S5).

To assess the effects on glucose homeostasis, we performed glucose tolerance and insulin tolerance tests. Compared with vehicle-treated control mice, GV-971-treated DIO mice exhibited lower blood glucose levels following glucose challenge, improved insulin sensitivity, reduced fasting insulin concentrations, and decreased homeostatic model assessment of insulin resistance (HOMA-IR) indices (Fig. 1e–g). These findings indicate that GV-971 mitigates HFD-induced obesity and insulin resistance.

Consistent with the improvement in systemic metabolism, serum total cholesterol levels, liver weight, and hepatic triglyceride levels were reduced in GV-971-treated mice (Fig. 1h, i). Histological examination revealed fewer hepatic lipid droplets and an absence of overt cholestasis, inflammation, or necrosis after GV-971 treatment (Fig. 1j, k), suggesting protection against HFD-induced hepatic steatosis. In addition, serum alanine aminotransferase (ALT) and aspartate aminotransferase (AST) levels were decreased but not increased (Supplementary Fig. S1b), indicating that GV-971 was non-hepatotoxic and instead alleviated hepatic injury.

To explore the mechanism underlying GV-971-induced weight reduction, energy balance analysis revealed that GV-971 increased total energy expenditure without affecting food intake (Fig. 1l, m). At the molecular level, GV-971 upregulated the thermogenic gene *Ucp1* in subcutaneous white adipose tissue (Fig. 1n), whereas the expression of the other thermogenic regulators, namely, *Pgc1a, Prdm16, Adrb3, Dio2*^22,23^ and *Fgf21*^24,25^, remained largely unchanged (Supplementary Fig. S1c–h). Histological analysis revealed enhanced browning of inguinal white adipose tissue, characterized by the appearance of multilocular adipocytes (Fig. 1o), a hallmark of beige adipocyte formation^24,26^.

Collectively, these results demonstrate that GV-971 attenuates obesity and improves metabolic function in DIO mice.

### GV-971 modulates the composition of the gut microbiota in DIO mice

To investigate the impact of GV-971 on the gut microbial composition, the V3–V4 variable region of bacterial 16S rRNA from fecal samples was sequenced using the Illumina-MiSeq platform. Principal coordinate analysis (PCoA) of weighted UniFrac distances demonstrated clear segregation of microbial communities among the control, DIO, and GV-971-treated DIO groups, indicating that GV-971 markedly reshaped the gut microbiota composition (Supplementary Fig. S2a). At the phylum level, *Firmicutes* dominated the microbiota of DIO mice, whereas its relative abundance decreased following GV-971 treatment (Supplementary Fig. S2b). Linear discriminant analysis effect size (LEfSe) analysis revealed that *Clostridium* was a characteristic genus enriched in the DIO group, whereas its abundance decreased in GV-971-treated mice (Supplementary Fig. S2c, d). Collectively, these findings indicate that GV-971 modulates the gut microbial richness and community structure in DIO mice, notably by reducing the abundance of *Clostridium* populations within the phylum *Firmicutes*, thus restoring the microbial profile to a profile more similar to that of lean controls.

### The gut microbiota mediates the metabolic benefits of GV-971

**Fig. 2:**
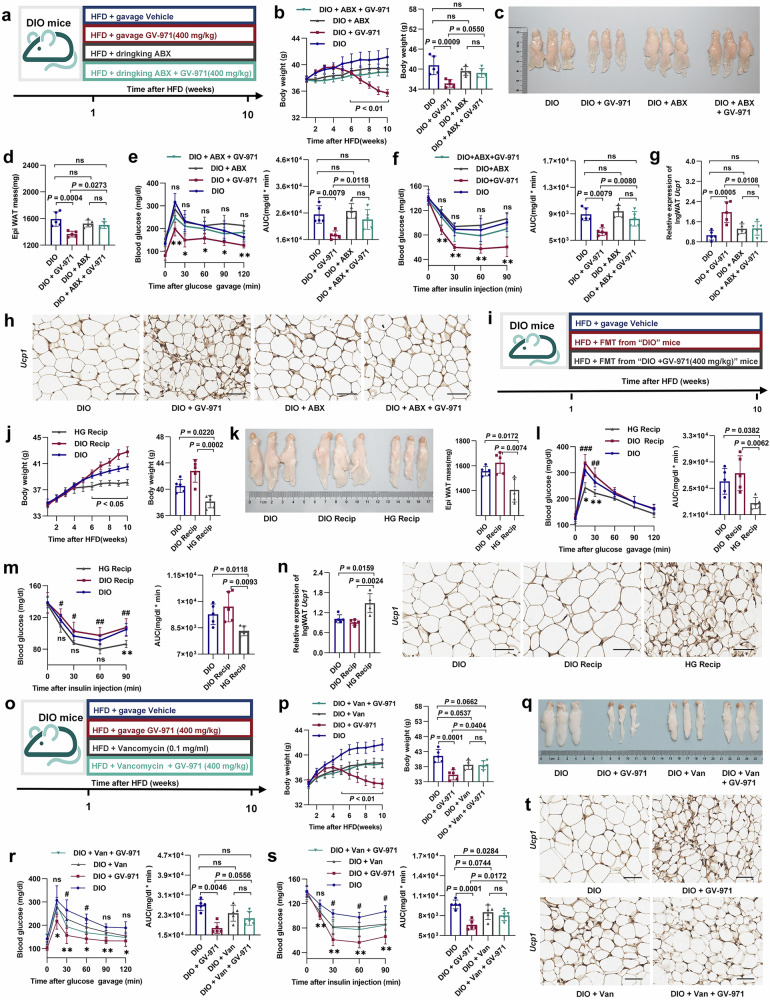
The gut microbiota mediates the effects of GV-971 on obesity and metabolic syndrome in DIO mice. **a** Schematic of the experimental procedure in **b**–**h**: DIO mice were classified into four groups: DIO mice treated with vehicle (DIO, *n* = 5), DIO mice treated with GV-971 (DIO + GV-971, *n* = 5), DIO mice treated with ABX (DIO + ABX, *n* = 5), and DIO mice treated with ABX and GV-971 (DIO + ABX + GV-971, *n* = 5). **b** Growth curves of body weight (left) and a statistical chart of the difference in body weight at week 10 (right). **c** Representative epididymis white adipose tissue (WAT). **d** Mass of the epididymis (Epi) WAT. **e** Glucose tolerance test. **f** Insulin tolerance test. **g** mRNA expression of *Ucp1* in inguinal (Ing) WAT. **h** Representative Ucp1 immunohistochemical staining of inguinal WAT. Scale bars: 50 μm. **i** Schematic of the experimental procedure in **j**–**n**: DIO mice were classified into three groups: DIO mice with vehicle (DIO, *n* = 5), DIO mice that received FMT from the DIO group (DIO recipient, *n* = 5) and DIO mice that received FMT from the HG group (HG recipient, *n* = 5). **j** Growth curves of body weight (left) and a statistical chart of the difference in body weight at week 10 (right). **k** Representative epididymal WAT (left) and mass of the epididymal WAT (right). **l** Glucose tolerance test. **m** Insulin tolerance test. **n** mRNA expression of *Ucp1* in inguinal WAT (left) and representative Ucp1 immunohistochemical staining of inguinal WAT (right). Scale bars: 50 μm. **o** Schematic of the experimental procedure in **p**–**t**: DIO mice were classified into four groups: DIO mice treated with vehicle (DIO, *n* = 5), DIO mice treated with GV-971 (DIO + GV-971, *n* = 5), DIO mice treated with vancomycin (DIO + Van, *n* = 5), and DIO mice treated with vancomycin and GV-971 (DIO + Van + GV-971, *n* = 5). **p** Growth curves of body weight (left) and a statistical chart of the difference in body weight at week 10 (right). **q** Representative epididymal WAT. **r** Glucose tolerance test. **s** Insulin tolerance test. **t** Representative Ucp1 immunohistochemical staining of inguinal WAT. Scale bars: 50 μm.

To determine whether the anti-obesity and metabolic dysfunction effects of GV-971 are mediated through the gut microbiota, DIO mice were treated with a broad-spectrum antibiotic cocktail (ABX) to deplete intestinal microbes (Fig. 2a). Microbiota depletion abolished the metabolic benefits of GV-971, as evidenced by the loss of its effects on body weight reduction (Fig. 2b), decreased fat mass (Fig. 2c, d), and improved glucose tolerance and insulin sensitivity (Fig. 2e, f). In addition, serum total cholesterol levels, liver weight, and hepatic triglyceride content were no longer reduced in GV-971-treated DIO mice following ABX treatment (Supplementary Fig. S3a, b). Histological analysis revealed that persistent hepatic lipid accumulation was accompanied by cholestasis, inflammation, and necrosis (Supplementary Fig. S3c, d), whereas ALT and AST levels remained elevated (Supplementary Fig. S3e), indicating that microbiota depletion negated GV-971-mediated hepatoprotection. At the molecular level, *Ucp1* expression in subcutaneous (inguinal) white adipose tissue was not significantly upregulated in ABX-treated mice (Fig. 2g), and histological analysis revealed no evidence of adipocyte browning (Fig. 2h).

To test whether GV-971-induced microbial alterations directly contribute to its metabolic benefits, we performed FMT using the microbiota from high-dose GV-971-treated DIO mice (HG donors) or untreated DIO mice (DIO donors) in antibiotic-cleared recipients (Fig. 2i). Compared with DIO microbiota, transplantation of HG microbiota reduced body weight gain (Fig. 2j), decreased fat mass (Fig. 2k), and improved glucose tolerance and insulin sensitivity (Fig. 2l, m). Moreover, HG microbiota transplantation decreased serum cholesterol levels, liver weight, triglyceride content (Supplementary Fig. S3f–h) as well as hepatic lipid droplet accumulation without evidence of cholestasis, inflammation, or necrosis (Supplementary Fig. S3i). Serum ALT and AST levels were also reduced (Supplementary Fig. S3j). Consistent with these improvements, *Ucp1* expression was upregulated, and beige adipocyte formation was evident in inguinal white adipose tissue (Fig. 2n). Thus, these findings demonstrate that GV-971-induced microbial remodeling directly mediates the alleviation of obesity and metabolic dysfunction in DIO mice.

To further elucidate the microbial drivers of these effects, the abundance of *Clostridium*, a genus markedly reduced by GV-971 treatment within the phylum *Firmicutes*, was selectively depleted using vancomycin^27^ (Fig. 2o). Vancomycin administration abolished the metabolic benefits of GV-971, with no significant differences observed in body weight, adiposity, glucose tolerance, or hepatic lipid accumulation between the DIO + vancomycin and DIO + vancomycin + GV-971 groups (Fig. 2p–t; Supplementary Fig. S4a–i).

Together, these data establish that the gut microbiota, particularly *Clostridium* species within *Firmicutes*, is essential for the anti-obesity effects and metabolic improvements induced by GV-971 in DIO mice.

### *C. scindens* is essential for GV-971-induced metabolic improvements

To identify the bacterial species responsible for the GV-971-induced modulation of *Clostridium* populations, we analyzed the correlations between alterations in the gut microbiota and metabolic phenotypes. Metagenomic profiling revealed that *C. scindens* was enriched in DIO mice, whereas its abundance was reduced following GV-971 treatment (Supplementary Figs. S5a–c and S6a–c).

**Fig. 3:**
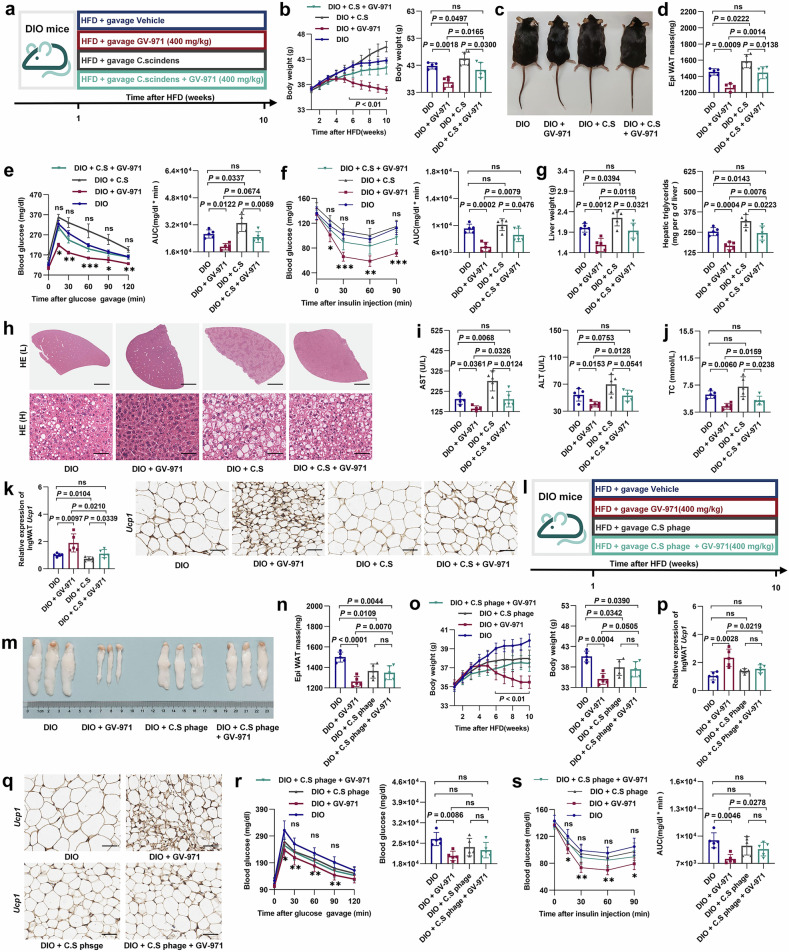
*C. scindens* mediates the effects of GV-971 on obesity and metabolic syndrome in DIO mice. **a** Schematic diagram of the experimental procedure in **b**–**k**: DIO mice were classified into four groups: DIO mice treated with vehicle (DIO, *n* = 5), DIO mice treated with GV-971 (400 mg/kg) (DIO + GV-971, *n* = 5), DIO mice that received *C. scindens* (DIO + C. S, *n* = 5), and DIO mice that received *C. scindens* and GV-971 (400 mg/kg) (DIO + C. S + GV-971, *n* = 5). **b** Growth curves of body weight (left) and a statistical chart of the difference in body weight at week 10 (right). **c** Representative mice from different groups. **d** Mass of the epididymis (Epi) white adipose tissue (WAT). **e** Glucose tolerance test. **f** Insulin tolerance test. **g** Liver weights (left) and liver triglyceride contents (right). **h** Representative H&E staining of liver sections. HE (L) scale bars: 2000 μm; other scale bars: 100 μm. **i** Serum AST and ALT levels. **j** Serum total cholesterol (TC) levels. **k** mRNA expression of *Ucp1* in inguinal (Ing) WAT (left) and representative Ucp1 immunohistochemical staining of inguinal WAT (right). Scale bars: 50 μm. **l** Schematic of the experimental procedure in **m**–**s**: DIO mice were classified into four groups: DIO mice treated with vehicle (DIO, *n* = 5), DIO mice treated with GV-971 (400 mg/kg) (DIO + GV-971, *n* = 5), DIO mice that received *C. scindens* phage (DIO + C. S phage, *n* = 5), and DIO mice received *C. scindens* phage and GV-971 (400 mg/kg) (DIO + C. S phage + GV-971, *n* = 5). **m** Representative epididymal WAT. **n** Mass of the Epi WAT. **o** Growth curves of body weight (left) and a statistical chart of the difference in body weight at week 10 (right). **p** mRNA expression of *Ucp1* in Ing WAT. **q** Representative Ucp1 immunohistochemical staining of inguinal WAT. Scale bars: 50 μm. **r** Glucose tolerance test. **s** Insulin tolerance test.

To determine whether *C. scindens* mediates the metabolic benefits of GV-971, DIO mice receiving GV-971 were colonized with *C. scindens* by oral gavage for 10 weeks (Fig. 3a). *C. scindens* colonization abolished the beneficial effects of GV-971, including its ability to suppress body weight gain (Fig. 3b, c), reduce fat mass (Fig. 3d), and improve glucose tolerance and insulin sensitivity (Fig. 3e, f). In *C. scindens*-colonized mice, GV-971 failed to decrease liver weight, hepatic triglyceride accumulation, or lipid droplet deposition, and histological analysis revealed persistent cholestasis, inflammation, and necrosis (Fig. 3g, h). Serum ALT, AST, and total cholesterol levels also remained elevated (Fig. 3i, j), confirming the loss of hepatoprotective effects. At the molecular level, *Ucp1* expression in subcutaneous (inguinal) white adipose tissue was not significantly induced, and browning of white adipose tissue was absent, as evidenced by the lack of multilocular adipocytes (Fig. 3k).

Pathologically, *C. scindens* colonization alone exacerbated obesity and metabolic impairments in DIO mice, suggesting a direct pathogenic contribution of this species. To further confirm its causal role, we administered *C. scindens*-specific bacteriophages to selectively deplete the bacterium in DIO mice (Fig. 3l; Supplementary Fig. S7a–c)^28^. Strikingly, depletion of *C. scindens* using phage therapy abolished the differential effects between GV-971-treated and untreated mice, with comparable outcomes in body weight, adiposity, glucose tolerance, and hepatic lipid metabolism across the DIO + *C. scindens* phage and DIO + *C. scindens* phage + GV-971 groups (Fig. 3m–s; Supplementary Fig. S7d–h).

Together, these data demonstrate that *C. scindens* is a pivotal microbial mediator of the effects of GV-971 and that its downregulation is essential for the GV-971-induced amelioration of obesity and metabolic dysfunction in DIO mice.

### GV-971-induced downregulation of DCA is correlated with reduced obesity and metabolic dysfunction in DIO mice

The impact of the gut microbiota on the host is predominantly mediated by bacterial metabolites^29–33^. To elucidate the mechanisms underlying the metabolic benefits of GV-971, non-targeted metabolomic profiling of microbiota-associated metabolites was performed on fecal samples from control, DIO, and high-dose GV-971-treated DIO mice. In total, 153 metabolites were identified, including organic acids, fatty acids, bile acids, amino acids, phenylpropionates, benzene derivatives, indoles, benzoic acids, SCFAs, carbohydrates, and other compounds. Principal component analysis (PCA) revealed distinct clustering among the three groups, indicating that GV-971 treatment markedly altered the intestinal metabolite landscape (Supplementary Fig. S8a).

At the metabolite class level, GV-971 increased the relative abundance of organic acids, carbohydrates, and indoles while reducing bile acid and SCFA levels in the cecal contents of DIO mice (Supplementary Fig. S8b). Subsequent differentially abundant metabolite screening (Supplementary Fig. S8c) revealed 135 altered metabolites, including five bile acids. Volcano plot analysis revealed that the concentration of DCA, a secondary bile acid derived from bacterial 7α-dehydroxylation, was greater in DIO mice than in control mice but significantly decreased following GV-971 treatment (Supplementary Figs. S8d and S9a–h).

Studies have clearly shown that the secondary bile acid DCA is significantly increased in the intestinal and fecal samples of HFD-induced obese mice^24,27,34–40^. To explore the relationship between bile acid alterations and metabolic outcomes, correlation analyses were performed between fecal DCA levels and metabolic indicators. The DCA concentration was strongly positively correlated with body weight, adiposity, insulin resistance, and hepatic steatosis (Supplementary Fig. S9i).

Thus, these findings suggest that GV-971 improves obesity and metabolic dysfunction in DIO mice, at least in part through the suppression of microbiota-derived DCA production.

### Supplementation with DCA attenuates the metabolic benefits of GV-971

**Fig. 4:**
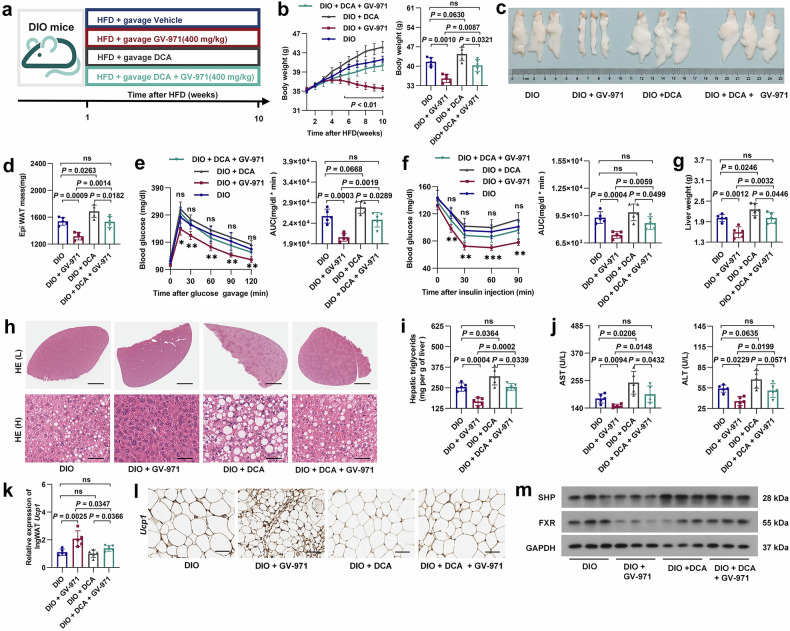
Replenishing DCA weakens the effects of GV-971 in alleviating the symptoms of obesity and metabolic dysfunction in DIO mice. **a** Schematic of the experimental procedure: DIO mice were classified into four groups: DIO mice treated with vehicle (DIO, *n* = 5), DIO mice treated with GV-971 (400 mg/kg) (DIO + GV-971, *n* = 5), DIO mice treated with DCA (DIO + DCA, *n* = 5), and DIO mice treated with DCA and GV-971 (400 mg/kg) (DIO + DCA + GV-971, *n* = 5). **b** Growth curves of body weight (left) and a statistical chart of the difference in body weight at week 10 (right). **c** Representative epididymal white adipose tissue (WAT) from the different groups. **d** Mass of the epididymis (Epi) WAT. **e** Glucose tolerance test. **f** Insulin tolerance test. **g** Liver weight. **h** Representative H&E staining of liver sections. HE (L) scale bars: 2000 μm; other scale bars: 100 μm. **i** Liver triglyceride contents. **j** Serum AST and ALT levels. **k** mRNA expression of *Ucp1* in inguinal (Ing) WAT. **l** Representative Ucp1 immunohistochemical staining of inguinal WAT. Scale bars: 50 μm. **m** Western blot analysis (cropping of blot images) of FXR and SHP protein expression in the intestine.

To determine whether DCA contributes to the metabolic improvements induced by GV-971, DIO mice receiving GV-971 were supplemented with DCA by oral gavage for 10 weeks (Fig. 4a). DCA supplementation abolished the beneficial effects of GV-971, eliminating its ability to suppress body weight gain (Fig. 4b), reduce fat mass (Fig. 4c, d), and improve glucose tolerance and insulin sensitivity (Fig. 4e, f). Similarly, GV-971 failed to reduce liver weight, hepatic triglyceride levels, or hepatic lipid accumulation in DCA-treated mice; instead, hepatic cholestasis, inflammation, and necrosis were evident (Fig. 4g–i). Moreover, serum ALT and AST levels remained elevated (Fig. 4j), and *Ucp1* expression in subcutaneous (inguinal) white adipose tissue was not upregulated (Fig. 4k). Histological examination revealed no evidence of white adipose tissue browning or multilocular adipocytes (Fig. 4l). These findings demonstrate that supplementation with DCA counteracts the metabolic benefits of GV-971.

While DCA serves as a natural agonist of intestinal FXR^27,41^, the bile acid receptor FXR plays a complex and dual role in metabolic regulation, where its site of action (liver vs intestine) and state of activation can lead to diverse outcomes^40^. To further explore the underlying mechanism, bile acid signaling molecules were analyzed in the liver and intestine^27,42–44^. Compared with that in the DIO group, intestinal FXR expression was downregulated in the GV-971-treated group, whereas the expression of other bile acid-responsive receptors, hepatic FXR, intestinal TGR5, PXR, and VDR, did not significantly change (Supplementary Fig. S10a–f). Functionally, supplementation with DCA counteracted the inhibitory effect of GV-971 on intestinal FXR expression (Fig. 4m). Together, these findings indicate that GV-971 restores metabolic homeostasis by decreasing DCA levels and attenuating intestinal FXR signaling.

### *EcN-baiF* administration abolishes the metabolic benefits of GV-971

To investigate whether the GV-971-induced reduction in *C. scindens* abundance was linked to a decrease in DCA levels, we analyzed the correlation between *Clostridium* species abundance and bile acid profiles. Correlation analysis revealed a strong positive correlation between *C. scindens* abundance and intestinal DCA concentration (Supplementary Fig. S11a). To further explore the underlying mechanism, we quantified the expression of bile acid conversion genes involved in the 7α-dehydroxylation pathway^33,38^. Among these genes, *baiF*, which encodes a key enzyme responsible for the conversion of primary to secondary bile acids^45–48^, was significantly downregulated in GV-971-treated DIO mice compared with vehicle-treated controls. In contrast, the bile salt hydrolase activity and the expression of other bile acid conversion genes — including *cgh, hdhA, baiA, baiB, baiCD, baiE, baiH*, and *baiI* — remained unchanged (Supplementary Fig. S11b, c).

**Fig. 5:**
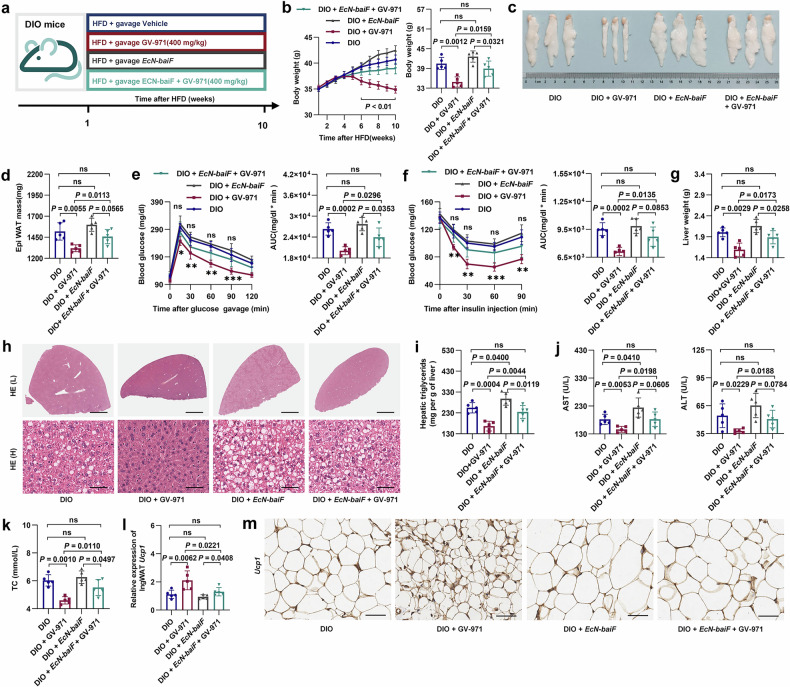
The engineered *EcN-baiF* reversed the alleviation of symptoms of obesity and metabolic dysfunction induced by GV-971. **a** Schematic of the experimental procedure: DIO mice were classified into four groups: DIO mice treated with vehicle (DIO, *n* = 5), DIO mice treated with GV-971 (400 mg/kg) (DIO + GV-971, *n* = 5), DIO mice treated with *EcN*-*baiF* (DIO + *EcN*-*baiF*, *n* = 5), and DIO mice treated with *EcN*-*baiF* and GV-971 (400 mg/kg) (DIO + *EcN*-*baiF* + GV-971, *n* = 5). **b** Growth curves of body weight (left) and a statistical chart of the difference in body weight at week 10 (right). **c** Representative epididymal white adipose tissue (WAT) from the different groups. **d** Mass of the epididymal (Epi) WAT. **e** Glucose tolerance test. **f** Insulin tolerance test. **g** Liver weight. **h** Representative H&E staining of liver sections. HE (L) scale bars: 2000 μm; other scale bars: 100 μm. **i** Liver triglyceride contents. **j** Serum AST and ALT levels. **k** Serum total cholesterol (TC) level. **l** mRNA expression of *Ucp1* in inguinal (Ing) WAT. **m** Representative Ucp1 immunohistochemical staining of inguinal WAT. Scale bars: 50 μm.

To determine whether *baiF* downregulation contributes causally to the metabolic improvements induced by GV-971, we constructed an engineered *Escherichia coli Nissle* 1917 strain expressing *baiF* (*EcN*-*baiF*) (Supplementary Fig. S11d–f). This engineered strain was orally administered to GV-971-treated DIO mice for 10 weeks (Fig. 5a). *EcN*-*baiF* colonization completely abolished the metabolic benefits of GV-971, including its effects on body weight reduction (Fig. 5b, c) and fat mass decrease (Fig. 5d), and improved glucose tolerance and insulin sensitivity (Fig. 5e, f).

Consistent with these findings, liver weight, hepatic triglyceride content, and lipid droplet accumulation were not reduced by GV-971 in *EcN*-*baiF*-colonized mice (Fig. 5g–i), and hepatic cholestasis, inflammation, and necrosis persisted (Fig. 5h). Similarly, serum ALT, AST, and total cholesterol levels remained elevated (Fig. 5j, k), whereas *Ucp1* expression and browning of subcutaneous (inguinal) white adipose tissue were absent (Fig. 5l, m).

These findings demonstrate that GV-971 improves metabolic dysfunction in DIO mice by reducing the abundance of *C. scindens*, which expresses *baiF* and 7α-dehydroxylase.

### Intestinal FXR is required for GV-971-induced metabolic improvements

**Fig. 6:**
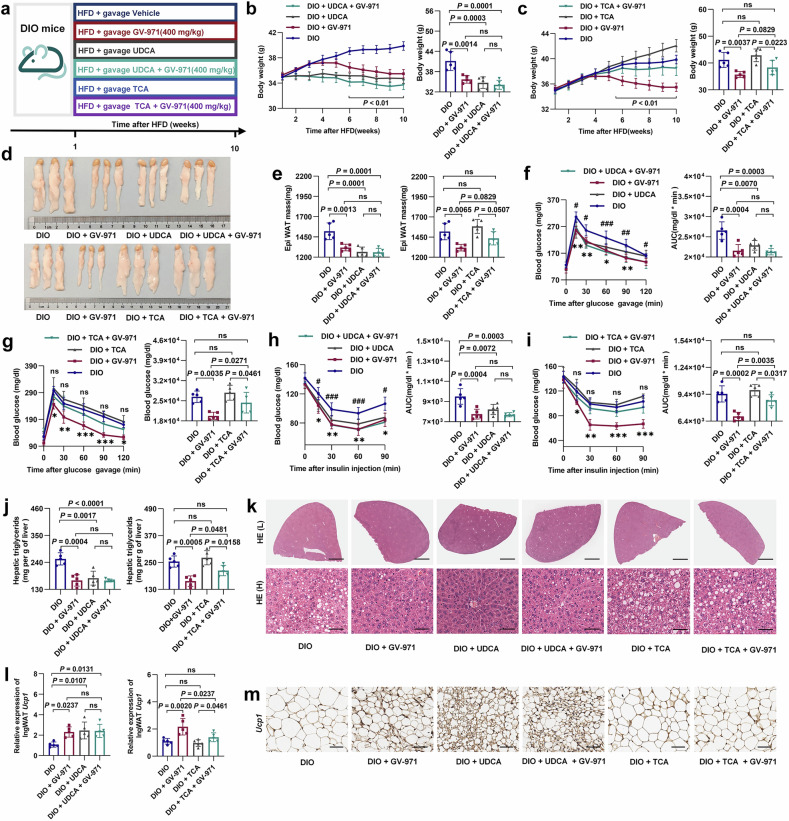
Intestinal FXR signaling is essential for the long-term GV-971-induced alleviation of symptoms of obesity and metabolic dysfunction in DIO mice. **a** Schematic of the experimental procedure: DIO mice were classified into six groups: DIO mice treated with vehicle (DIO, *n* = 5), DIO mice treated with GV-971 (400 mg/kg) (DIO + GV-971, *n* = 5), DIO mice treated with UDCA (DIO + UDCA, *n* = 5), DIO mice treated with UDCA and GV-971 (400 mg/kg) (DIO + UDCA + GV-971, *n* = 5), DIO mice treated with TCA (DIO + TCA, *n* = 5), and DIO mice treated with TCA and GV-971 (400 mg/kg) (DIO + TCA + GV-971, *n* = 5). **b**, **c** Body weight growth curves (left) and a statistical chart of the difference in body weight at week 10 (right). **d** Representative epididymal white adipose tissue (WAT) from the different groups. **e** Mass of the epididymal (Epi) WAT. **f**, **g** Glucose tolerance test. **h**, **i** Insulin tolerance test. **j** Liver triglyceride contents. **k** Representative H&E staining of liver sections. HE (L) scale bars: 2000 μm; other scale bars: 100 μm. **l** mRNA expression of *Ucp1* in inguinal (Ing) WAT. **m** Representative Ucp1 immunohistochemical staining of inguinal WAT. Scale bars: 50 μm.

Accumulating evidence suggests that the inhibition of intestinal FXR signaling can improve metabolism^38,39,49–52^. The underlying mechanism is related to the inhibition of the excessive activation of intestinal FXR, which can ameliorate metabolic diseases and obesity via the intestinal FXR-ceramide axis^27,40^. To determine whether intestinal FXR mediates the metabolic benefits of GV-971, we treated DIO mice with the intestinal FXR agonist taurocholic acid (TCA) or the endogenous inhibitor ursodeoxycholic acid (UDCA)^38^, followed by a 10-week course of treatment with GV-971 (Fig. 6a). In TCA-treated mice, compared with TCA alone, GV-971 restricted body weight gain, reduced fat mass, and improved glucose tolerance and insulin sensitivity (Fig. 6b–i). In contrast, co-administration of UDCA abolished these effects, as compared with UDCA alone, GV-971 failed to improve metabolic parameters (Fig. 6b–i).

Consistent with these findings, GV-971 treatment in TCA-supplemented DIO mice significantly reduced liver weight, hepatic triglyceride accumulation, and hepatic lipid droplet accumulation, whereas no such changes were observed in UDCA-treated mice (Fig. 6j, k). GV-971 also decreased serum ALT, AST, and total cholesterol levels in the TCA group but not in the UDCA group (Supplementary Fig. S12a–c).

At the adipose tissue level, GV-971 upregulated thermogenic gene expression in subcutaneous white adipose tissue and enhanced Ucp1-mediated beige adipocyte formation in TCA-treated mice (Fig. 6l, m). However, these effects were absent in UDCA-treated animals. These results indicate that intestinal FXR is required for GV-971-induced metabolic improvements.

## Discussion

By integrating multi-omics profiling with targeted genetic and microbial interventions, this study elucidates a mechanistic framework through which GV-971 confers robust protection against HFD-induced obesity and metabolic dysfunction. We identified a distinct bacterium-metabolite-host signaling axis in which GV-971 remodels the gut microbiota to suppress *C. scindens*-mediated bile acid metabolism, leading to decreased intestinal DCA production and attenuated FXR activation. This axis links microbiota-derived metabolites to host metabolic homeostasis, establishing a paradigm in which a pharmacological agent reprograms gut ecology to achieve systemic metabolic benefit.

A major conceptual advance of this study is the transition from correlation to causality in microbiome‒host interactions. Through loss- and gain-of-function strategies, such as antibiotic depletion, bacteriophage targeting, and microbial reconstitution, we demonstrate that *C. scindens* acts as a keystone species that drives metabolic impairment and that its suppression is essential for GV-971 activity. These manipulations confirm that *C. scindens* is not merely a biomarker of dysbiosis but also an active metabolic “pathobiont” whose modulation can reshape host physiology.

Mechanistically, our findings converge on secondary bile acid metabolism. The identification of DCA as the key effector metabolite downstream of *C. scindens* and upstream of intestinal FXR provides a biochemical explanation for the metabolic benefits of GV-971. Complementary gain-of-function interventions — exogenous DCA supplementation and colonization with *EcN-baiF*, an engineered bacterium overexpressing the bile acid-converting enzyme gene *baiF*, recapitulated metabolic deterioration and abolished the efficacy of GV-971. These results suggest that suppression of *baiF*-dependent 7α-dehydroxylation and a reduction in DCA levels are central to the effects of GV-971. While targeted genetic manipulation of *C. scindens* remains technically challenging, our heterologous expression of *baiF* in *E. coli* Nissle provides compelling gain-of-function evidence that this enzyme is not only necessary but also causally sufficient to mediate the therapeutic effects of GV-971.

**Fig. 7:**
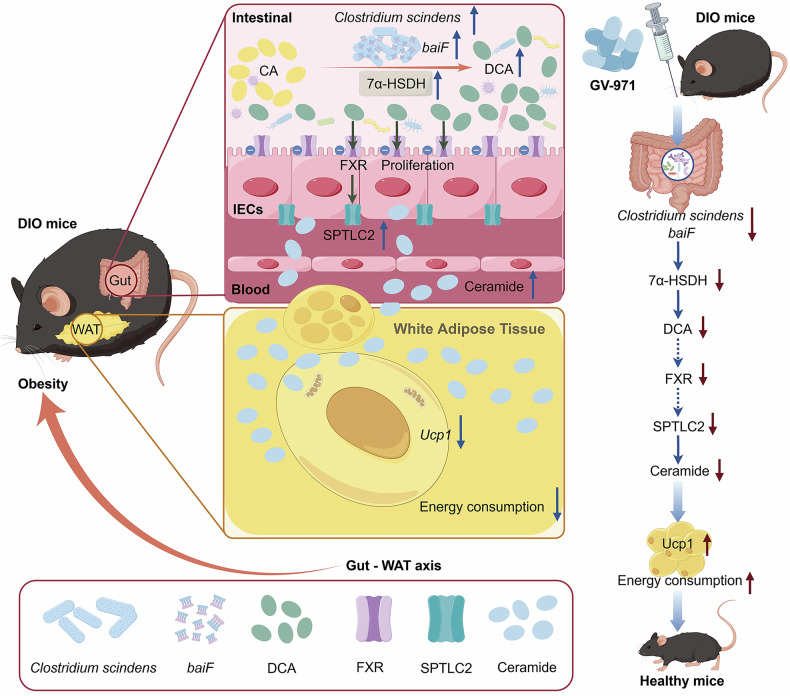
Schematic depicting the therapeutic effects of GV-971 on obesity and metabolic dysfunction. GV-971 remodels the gut microbiota, resulting in a specific reduction in the abundance of *Clostridium scindens*, a pivotal bacterium involved in secondary bile acid metabolism. This reduction leads to the downregulation of the bacterial gene *baiF*, which encodes the enzyme 7α hydroxysteroid dehydrogenase (7α-HSDH), which is essential for DCA production. Consequently, the level of DCA in the intestinal lumen is decreased, alleviating the excessive activation of intestinal FXR. This suppression of intestinal FXR signaling confers systemic metabolic benefits via the gut FXR-ceramide axis. This signaling cascade promotes the expression of the thermogenic gene *Ucp1* in white adipose tissue (WAT) and WAT browning, ultimately ameliorating obesity and metabolic dysfunction.

The physiological context of these findings aligns with emerging evidence that intestinal FXR inhibition exerts metabolic benefits^38,39,49–52^. While hepatic FXR activation contributes to bile acid homeostasis^40^, intestinal FXR hyperactivation promotes ceramide synthesis and insulin resistance^38,39^. By decreasing the level of luminal DCA, a potent intestinal FXR agonist, GV-971 acts as an indirect intestinal FXR inhibitor (Supplementary Figs. S13a–e and S14a–b), thus restoring metabolic balance through the gut FXR-ceramide axis (Fig. 7; Supplementary Fig. S15a–f). This microbiota-derived, localized modulation of FXR signaling offers a physiologically selective alternative to systemic FXR antagonists, which often cause dyslipidemia and other adverse effects.

It is important to contextualize our findings with those of a previous study in which intestinal FXR activation promoted adipose tissue browning and alleviated obesity^53^. While these results may contradict our conclusion that intestinal FXR inhibition is metabolically beneficial, the divergence likely reflects the complex, context-dependent nature of FXR signaling. The metabolic outcomes of FXR modulation may depend on several factors, including the mode of intervention (genetic overexpression vs pharmacological inhibition), the specific chemical nature of the ligand (synthetic agonist GW4064 vs bile acid metabolism), and the baseline metabolic state of the animal. It is plausible that intestinal FXR operates within a homeostatic window; either extreme activation or inhibition could trigger compensatory mechanisms that ultimately converge on improved energy expenditure or reduced adiposity, albeit through distinct molecular pathways (e.g., the CerS6 axis vs the ceramide axis). Future studies are warranted to dissect the conditions under which FXR agonism or antagonism favors metabolic health. In addition, this study demonstrated only from a pharmacological perspective the significance of the intestinal FXR in the metabolic improvement induced by GV-971. In future studies, more in-depth studies from the perspective of genetics are needed.

Notably, while Wu et al. reported that exogenous DCA promotes thermogenesis by activating TGR5 in white adipose tissue, leading to the upregulation of *Ucp1* and *Ckmt2* expression^54^, this study revealed that elevated intestinal DCA levels instead inhibit *Ucp1*‑mediated adipose browning through the activation of the intestinal FXR‑ceramide axis. This apparent discrepancy may be attributed to several key factors. First, the concentration gradients and differential receptor affinities of DCA likely play a decisive role. Under physiological or pharmacological conditions, DCA activates TGR5 and FXR in a concentration‑dependent manner: lower concentrations of DCA (typically in the micromolar range) preferentially bind to TGR5^55,56^, whereas higher concentrations, especially when locally accumulated in the intestinal lumen, tend to activate FXR^57^. The exogenous DCA regimen used by Wu et al. likely resulted in moderate levels of circulating DCA, which preferentially acts on adipose TGR5. In contrast, in our model, diet-induced obesity led to a marked local increase in DCA in the intestinal epithelium, which was sufficient to efficiently activate intestinal FXR but not necessarily to proportionally increase circulating DCA levels. Thus, differences in the site of action (intestinal local vs systemic circulation) and concentration thresholds may explain why DCA produces opposite thermogenic effects via the intestinal FXR axis vs the adipose TGR5 axis. Second, cell type‑specific signal integration should not be overlooked. Ceramide generation following intestinal FXR activation and its subsequent transport to adipose tissue may directly counteract TGR5‑mediated thermogenic gene expression by inhibiting the cAMP/PGC1α pathway in adipocytes^58^. Moreover, ceramides themselves can promote lysosomal degradation of UCP1^59^, further attenuating the transcriptional upregulation by TGR5. Therefore, when DCA simultaneously exposes both the intestine and adipose tissue, the intestinal FXR‑ceramide axis may exert a dominant inhibitory effect on adipose thermogenesis, whereas the effect of TGR5 becomes apparent only in the absence of this gut‑adipose crosstalk (e.g., when exogenous DCA is directly administered peripherally). Third, differences in experimental systems include the spatiotemporal specificity of genetic modification (intestinal epithelial HIF‑2α knockout vs systemic exogenous DCA administration), the kinetics of DCA exposure (chronic endogenous accumulation vs acute exogenous injection), and the ambient temperature and metabolic state (cold exposure vs room temperature housing). These factors can affect the expression of bile acid-metabolizing enzymes (e.g., CYP7A1 and CYP27A1) and the composition of the gut microbiota^33,52^, thus altering DCA production rates and the profile of secondary metabolites. In summary, we propose that the bidirectional regulation of thermogenesis by DCA reflects a complex balance between intestinal local concentration, receptor selectivity, and the interplay of the gut‑adipose axis vs direct peripheral effects. Future studies using tissue‑specific FXR or TGR5 knockout models, combined with graded DCA infusions and stable isotope tracing, will be necessary to precisely elucidate this concentration‑dependent inter‑organ communication mechanism.

In addition to providing mechanistic insights, our findings have broad translational implications. At the fundamental level, this work delineates a causal chain linking a small-molecule drug to specific microbes, microbial metabolites, and host signaling pathways, offering a model for mechanistic microbiome research. Clinically, this suggests an opportunity for drug repurposing, as GV-971 already possesses established human safety data. Moreover, the database analysis revealed that the abundance of *Firmicutes* and *Clostridium* in obese patients was significantly greater than that in healthy individuals (Supplementary Fig. S16a–c). Consistent with these findings, the 16S rRNA sequencing results indicated that GV-971 treatment significantly reduced the abundance of *Firmicutes* and *Clostridium* in obese mice to the same level as that in normal mice (Supplementary Fig. S16d). On the basis of these findings, we further performed metagenomic sequencing on representative samples to achieve more detailed, species-level identification of *Clostridium*. The metagenomic data not only validated the shifts in *Clostridium* abundance observed with 16S rRNA sequencing but also enabled the precise identification of specific *Clostridium* species. The outcomes of these two sequencing approaches are complementary and consistent; 16S rRNA sequencing provided a broad overview of the community structure, whereas metagenomic sequencing offered a deeper, species-level resolution of the key species *C. scindens*, collectively supporting the conclusions of this study.

To investigate whether GV-971 reduces the abundance of *C. scindens* through direct antibacterial activity or by reshaping the intestinal microbial community, a network analysis, potential functional analysis, and in vitro bacterial culture experiments were conducted on the basis of the microbial community. The network analysis results revealed that the dominant bacteria in the obese mice (DIO), *Clostridium*, exhibited a co-exclusion relationship with the subordinate bacteria, *Lactobacillus*, whereas in the normal mice (Ctr), the dominant bacteria, such as *Lactobacillus*, *Bifidobacterium* and *Akkermansia*, showed a co-occurrence relationship. Moreover, in obese mice treated with GV-971 (HG), *Lactobacillus*, *Bifidobacterium* and *Akkermansia* became the dominant bacteria (Supplementary Fig. S17a). The potential functional analysis indicated that GV-971 might primarily change the intestinal microbial community through its participation in bacterial metabolism (carbohydrate metabolism) (Supplementary Fig. S17b). The in vitro bacterial culture results revealed that GV-971 promoted the growth of *Lactobacillus* (Supplementary Fig. S18a, b) but did not significantly promote or inhibit the growth of *C. scindens* (Supplementary Fig. S18c). Therefore, the results of in vitro bacterial culture experiments indicate that rather than reducing its abundance through direct antibacterial activity, GV-971 mainly reshapes the intestinal microbial community to reduce the abundance of *C. scindens* in obese mice. Further in-depth analysis of these results suggested that the remodeling effect of GV-971 on the intestinal microbial community might involve a more complex mechanism. The results of the network analysis revealed that the co-occurrence or antagonistic relationships between the dominant bacteria in different groups of mice changed significantly. These findings may indicate that GV-971 not only promotes or inhibits the growth of certain bacteria but also regulates the relationships between microbial members to change the community structure. The potential functional analysis revealed that GV-971 participates in bacterial carbohydrate metabolism, which may be key for its ability to remodel the intestinal microbial community. Carbohydrates are important energy sources for intestinal microorganisms, and GV-971 may affect the metabolism of carbohydrates in bacteria, thus changing the living environment and competitive situation of different bacteria. For example, it may enable some originally subordinate probiotics, such as *Lactobacillus*, *Bifidobacterium*, and *Akkermansia*, to gain growth advantages in the new carbohydrate metabolism environment and become dominant bacteria.

However, the optimal dosage and treatment duration of GV-971 remain to be determined. In this study, although higher doses produced progressively stronger effects over time, the incremental benefit beyond 200 mg/kg was minimal, with the 400 mg/kg group reaching a plateau from week 18 onward (Fig. 1). The wide confidence intervals and susceptibility to outliers underscore the need for larger, adequately powered studies to more precisely characterize the dose-response relationship. Future studies may therefore consider reducing the high dose or shortening the treatment duration. Mechanistic investigations should further elucidate how GV-971 modulates bacterial carbohydrate metabolism, for example, by applying gene-editing approaches to assess changes in bacteria-related gene expression and to identify specific metabolic targets. In addition, the results of human clinical trials can verify the remodeling and safety-related effects of GV-971 on the intestinal microbial community in humans, providing a more solid basis for its use in the clinical treatment of obesity and related diseases.

Conceptually, this study supports a precision microbiome-modulation strategy, demonstrating that systemically administered drugs can target keystone microbial species to achieve therapeutic benefit. Moreover, the identified biomarkers, *C. scindens* abundance, DCA levels, and *baiF* expression, may serve as predictors of treatment responsiveness, paving the way for personalized intervention. However, the translation of these findings from mice to humans requires careful validation as microbial ecology, bile acid metabolism, and diet differ substantially between species. In summary, this study defines a microbiota-bile acid-FXR signaling axis as the mechanistic basis of the metabolic benefits of GV-971 and exemplifies how drug-mediated microbial remodeling can reprogram host metabolism. These insights establish a foundation for developing precision microbiome-targeted therapeutics that harness existing pharmacological agents to treat complex metabolic diseases.

## Materials and Methods

### Animal studies

All animal experiments were performed in accordance with guidelines approved by the Animal Ethics Committee of Guangzhou Medical University (GY-2024-652). All the mice were housed in a standard specific-pathogen-free environment. For the DIO model, 6–8-week-old male C57BL/6J mice were fed an HFD (BIOPIKE, Cat# D12492) for 10 weeks. For the GV-971 treatment experiment, the DIO mice were orally gavaged with GV-971 at low (100 mg/kg/day, LG), medium (200 mg/kg/day, MG), and high (400 mg/kg/day, HG) dosages (Green Valley, Shanghai, China) dissolved in water under HFD conditions for 15 weeks.

To determine the role of GV-971 in improving obesity and metabolic dysfunction in DIO mice through the regulation of the gut microbiota, DIO mice received a daily oral gavage (200 μL/mouse) of an ABX consisting of metronidazole (200 mg/kg), neomycin sulfate (200 mg/kg), ampicillin (200 mg/kg) and vancomycin (100 mg/kg) for 3 days (Cat# A600633, Cat# A610366, Cat# A610028 and Cat# A600983, respectively; Sangon, Shanghai, China). Afterward, the DIO mice received drinking water containing an antibiotic cocktail (neomycin (1 mg/mL), streptomycin (1 mg/mL), and bacitracin (1 mg/mL)). The antibiotic-containing water was replaced every other day. For the gut microbiota depletion experiment, DIO mice were fed an HFD supplemented with GV-971 (400 mg/kg) for 10 weeks. For the gut microbiota transplantation experiments^21,38^, feces samples were collected from DIO mice with and without GV-971 treatment (referred to as “DIO Recip” and “(DIO + GV-971 (400 mg/kg)) Recip”). The feces samples were homogenized in PBS supplemented with 20% glycerin in a 50 mL tube (20 mg feces/1 mL liquid). The tube was then centrifuged (100× *g*, 10 min), and the whole supernatant was aliquoted and frozen at –80 °C. The mice were fed an HFD and administered 200 μL of the above-described supernatant by gavage after 3 days of antibiotic treatment.

In a model deficient in *Clostridium* species, the effects of *Clostridium* species were specifically inhibited by the use of vancomycin (0.1 mg/mL), and obesity and metabolic phenotypes were tested after DIO mice deficient in *Clostridium* species were treated with GV-971 (400 mg/kg) for 10 weeks to verify that GV-971 did not improve obesity or metabolic dysfunction in the DIO mice deficient in *Clostridium* species.

To transplant *C. scindens*, we fed DIO mice *C. scindens* (10^8^ CFU/mouse) by gavage for 10 weeks. Additionally, the mice were given *C. scindens* (10^8^ CFU/mouse) or GV-971 (400 mg/kg) plus *C. scindens* (10^8^ CFU/mouse) by gavage on an HFD for 10 weeks to explore the reversal effects.

To further investigate whether the ability of GV-971 to improve obesity and metabolic dysfunction in DIO mice was indeed mediated by *C. scindens*, a lytic bacteriophage that specifically targets *C. scindens* from fecal samples was isolated. For the *C. scindens* phage experiments, DIO mice were given *C. scindens* phage (8 × 10^9^ PFU/mouse) or GV-971 (400 mg/kg) plus *C. scindens* phage (8 × 10^9^ PFU/mouse) by oral gavage every 2 days on an HFD for 10 weeks.

In the DCA (Sigma‒Aldrich, Cat# 30960) recompensation experiment, DIO mice were given vehicle or GV-971 (400 mg/kg) in the presence of DCA (40 mg/kg) under HFD conditions for 10 weeks to explore the reversal effects^60^.

Engineered *EcN*-*baiF* was constructed to further verify that GV-971-induced improvements in obesity and metabolic syndrome are indeed mediated by the *baiF* gene, which encodes 7α-dehydroxylation enzymes responsible for converting primary bile acids to secondary bile acids. DIO mice were given *EcN*-*bai*F (10^8^ CFU/mouse) or GV-971 (400 mg/kg) plus *EcN*-*baiF* (10^8^ CFU/mouse) by gavage on an HFD for 10 weeks to explore the reversal effects.

To validate the inhibitory effects of GV-971 on intestinal FXR signaling, DIO mice were given vehicle or GV-971 (400 mg/kg) in the presence of UDCA (Sigma‒Aldrich, Cat# U5127) (50 mg/kg) or TCA (Sigma‒Aldrich, Cat# T4009) (50 mg/kg) under HFD conditions for 10 weeks.

### Metabolic assays

To assess glucose tolerance, the mice were fasted for 12 h, blood was drawn, and the mice were gavaged with 2 g/kg glucose. Blood samples were taken from the tail at 15 min, 30 min, 60 min, 90 min, and 120 min after gavage, and the glucose concentration was measured using a Roche superior blood glucose meter (Accu-Chek Performa). To ensure insulin tolerance, the mice were fasted for 4 h, blood was drawn, and then they were injected with insulin (Eli Lilly and Company) intraperitoneally at a dose of 1 U/kg body weight. Blood samples were taken from the tail at 15 min, 30 min, 60 min, and 90 min after injection, and the glucose concentration was measured using a Roche superior blood glucose meter (Accu-Chek Performa).

### Histological analysis

H&E staining was performed on formalin-fixed, paraffin-embedded sections using a standard protocol. Adipose tissues were fixed in 4% paraformaldehyde, and paraffin sections were cut and stained by immunostaining with a UCP1 (Abcam, Cat# ab10983) antibody. At least three discontinuous tissue sections were evaluated for each mouse.

### Triglyceride contents

Hepatic lipids were extracted using a 2:1 chloroform–methanol solution. Liver triglycerides were measured with a triglyceride colorimetric assay kit according to the manufacturer’s recommendation (Bioassay Systems, Hayward, CA, USA).

### RNA analysis

Adipose, liver, and intestinal tissues were frozen in liquid nitrogen and stored at –80 °C until RNA was prepared. RNA was extracted from frozen intestine and liver using TRIzol reagent (GeneKam Biotechnology AG). Complementary DNA was synthesized from 1 μg of total RNA using Superscript II reverse transcriptase (GeneKam Biotechnology AG). Quantitative PCR primers were designed with qPrimerDepot, and the sequences are shown in Supplementary Table S6. mRNA levels were normalized to those of the GAPDH gene and are expressed as the fold change relative to those of the control group.

### Western blot analysis

A 20–40 mg tissue sample was homogenized in RIPA buffer (containing a proteinase inhibitor mixture and phosphate buffer tablets; Roche, Indianapolis, USA) using a homogenizer. The protein concentration was determined using a BCA protein assay kit (Thermo Fisher Scientific, Waltham, MA, USA). In total, 20–30 μg of protein were separated by SDS-PAGE and then transferred onto a polyvinylidene fluoride membrane. The membrane was blocked for 1 h with a 5% (mass/volume) BSA (bovine serum albumin) solution dissolved in TBST (buffer) and then incubated with the primary antibody at 4 °C overnight. The membrane was then incubated with secondary antibody (Thermo Fisher Scientific, St. Louis, MO, USA) for 1 h. The signal was detected according to the manufacturer’s instructions (Thermo Fisher Scientific, Waltham, MA, USA).

### Serum biochemistry

Blood samples were obtained after 10 h of overnight fasting. The serum was obtained by centrifugation (4000× *g*, 10 min). TG, TCOH, ALT, and AST levels were detected using an automatic biochemical analyzer with a biochemical reagent (Kehua Bioengineering, Shanghai, China) at the Guangzhou Medical University Scientific Research Center.

### Profiling of the fecal microbiota

At the end of the experiment, fecal samples were collected from the mice and rapidly frozen in liquid nitrogen. They were then stored at –80 °C. These samples were carefully packaged on dry ice and sent to the laboratory of Metabo-Profile Biotechnology Co., Ltd. (Shanghai, China) for analysis. To extract total DNA from the fecal samples, we used the QIAamp Rapid DNA Mini Kit (from Qiagen Hilden, Germany) following the manufacturer’s recommended steps. After the DNA concentration and integrity assessment were completed, PCR amplification was performed on the 16S rRNA V3–V4 variable region (338 F: 5’-ACTCCTACGGGAGGCAGCA-3’; 806 R: 5’-GGACTACHVGGGTWTCTAAT-3’) to generate an amplicon sequencing library. The quality libraries were subsequently sequenced on the Illumina MiSeq platform following the manufacturer’s instructions. Microbiome bioinformatics analysis was performed using QIIME2 2019.4, with minor modifications based on the official tutorial (https://docs.qiime2.org/2019.4/tutorials/). In summary, the raw sequence data were demultiplexed using the demux plugin, followed by primer cutting with the cutadapt plugin. The sequences were then quality filtered, denoised, and merged and chimera were removed using the DADA2 plugin. Non-singleton amplicon sequence variants (ASVs) were aligned with mafft86 and used to construct a phylogenetic tree with fasttree2.87. The absolute abundance of ASVs was normalized using a standard of sequence numbers corresponding to the samples with the fewest sequences. Taxonomy was assigned to ASVs using the classify-sklearn naïve Bayes taxonomy classifier in the feature-classifier plugin88 against the SILVA database. Subsequent analyses of α-diversity and β-diversity were performed on the basis of the normalized output data. To analyze the diversity and richness of the communities in the sample, α-diversity was calculated from indices in QIIME2, including observed ASVs and Shannon analyses. PCoA using a Bray‒Curtis distance matrix calculation was followed by transformation to a new set of orthogonal axes. The first principal coordinate has the greatest variance, and the second principal coordinate has the second-largest variance. The results of the PCoA were displayed by the ade4 and ggplot2 packages in R software. In addition, beta-dispersion analysis using vegan betadisper was performed to evaluate the homogeneity of the group dispersions. To identify the differentially abundant microbial taxa between the experimental groups, LEfSe was performed within the QIIME2 software using the q2-LEfSe plugin. The samples were classified into predefined experimental groups on the basis of the relevant experimental conditions. Metadata were incorporated into the QIIME2 project for group assignment. Differentially abundant taxa were identified by applying a Kruskal‒Wallis test (α = 0.05) to detect features with significant differences in abundance across groups, followed by linear discriminant analysis (LDA) to estimate effect sizes, with an LDA score threshold of 2.0 or 3.0 (the specific threshold used is indicated in the figure legends) to identify biologically relevant features. For multiple comparisons, we applied Benjamini–Hochberg false discovery rate (FDR) correction to adjust the *P* values, ensuring the robustness of the results. The results were visualized using qiime LEfSe plot-res to generate bar plots of the LDA scores. Spearman correlation analysis was performed using GraphPad Prism. To further validate the differential abundance results, we conducted an Analysis of Composition of Microbiomes with Bias Correction (ANCOM-BC) using R.

### Microbial metabolism

At the end of the experiment, we collected fecal samples under sterile conditions, which were promptly frozen in liquid nitrogen and stored at –80 °C. These fecal samples, which were carefully packaged on dry ice, were then sent to the laboratory of Metabo-Profile Biotechnology Co., Ltd. (Shanghai, China) for subsequent analysis. A metabolomics analysis, as previously reported, was conducted on these fecal samples. First, the samples underwent homogenization and centrifugation, and the resulting supernatant was pooled. We employed an MPS2 multifunctional robot (Gerstel, Demilheim, NRW, Germany) for automated sample extraction and separation. Quantitative analysis of microbial metabolites was carried out using UPLC-MS/MS (Acquity UPLC, Xevo TQ-S, USA). In total, 201 standard substances from different chemical categories were prepared. Retention solutions were meticulously prepared for each compound of microbial metabolites. These solutions were based on methanol, ultrapure water, or sodium hydroxide solution. To ensure data quality and compensate for potential matrix effects, we used internal standards. The raw data files generated by UPLC-MS/MS were subsequently processed using TMBQ software (Shanghai, China). This software facilitated the integration, calibration, and quantification of each metabolite. For comprehensive analysis, various statistical techniques were employed, including PCA, PLS-DA, univariate analysis, and pathway analysis. These analyses were performed using a self-developed iMAP platform (v1.0, Metabo-Profile, Shanghai, China).

### Bacterial culture, transplantation and treatment

*C. scindens* was obtained from the American Type Culture Collection (ATCC, Cat# 35704, ATCC, MD, USA) and was anaerobically cultured in ATCC medium 2107. For bacterial transplantation in vivo, DIO mice were first treated with ABX for 1 week to deplete the gut microbiota under an HFD. *C. scindens* was orally administered 3 × 10^8^ CFU of bacteria/mouse twice per week for 10 weeks. Bacterial detection was confirmed by qPCR. The specific universal primers used are listed in Supplementary Table S6.

### *C. scindens* phage isolation and amplification

Phages targeting *C. scindens* were isolated from fecal samples. First, the fecal samples were resuspended in SM buffer (200 mM sodium chloride, 16 mM magnesium sulfate, and 0.1 M Tris-HCl, pH 7.4) and then centrifuged at 5000 rpm for 10 min at 4 °C to remove any residual solid matter. Afterward, the supernatant was filtered through 0.8 μm and 0.2 μm polyvinylidene fluoride filters. The treated samples were co-cultured with *C. scindens* three times. After the third enrichment, the supernatant was centrifuged at 12,000 rpm for 10 min at 4 °C and filtered through a 0.22 μm polyvinylidene fluoride filter. Overnight cultures of *C. scindens* (500 μL) were mixed with 4.5 mL of the upper layer of BHI agar (0.4% agar) and spread on the surface of the BHI agar plate (1.5% agar). After overnight incubation at 37 °C, 2.5 μL of the enriched droplet was spotted on the bacterial overlay plate to detect the presence of phages. The formed plaques were recovered using a sterile pipette tip in 500 μL of SM buffer. The phages were purified three times using the double-layer plate method to ensure that the phages were clonal isolates. By adding the above phage solution (2 mL) to 5 mL of *C. scindens* culture medium and then adding it to 45 mL of BHI medium, overnight culture could be performed at 37 °C. After the culture was completed, the lysate was centrifuged at 12,000 rpm and 4 °C for 10 min. Then, the supernatant was filtered through a 0.22 μm membrane filter, after which the titer of the phages was measured^28^.

### Construction and colonization of the *BaiF*-overexpressing *E. coli* Strain

*E. coli* Nissle 1917 (*EcN*) was purchased from ATCC (Manassas, VA, USA). The information and coding sequences of the *baiF* gene of *C. scindens* were extracted from the NCBI database, and these genes were optimized for expression in *EcN*. The region encoding the *baiF* enzyme was cloned and inserted into the sgRNA/Cas9 cloning plasmid and then introduced into *EcN* through heat stimulation to generate engineered bacteria (*EcN-baiF*). Colonies were selected on LB agar supplemented with kanamycin (50 µg/mL). Finally, pCP20 was utilized to eliminate the kanamycin resistance cassettes, resulting in the generation of the final antibiotic-free wild-type and *EcN-baiF* strains. Wild-type and *EcN-baiF* strains were cultured with LB and incubated on a shaker at 37 °C overnight. The wild-type and *EcN-baiF* strains were then harvested by centrifugation at 3000–5000× *g*, washed three times with sterile PBS, and resuspended in sterile ice-cold PBS (the final concentration of ~10^11^ CFU/mL).

### Bile acid conversion gene quantification

Fecal samples were collected from the mice and stored at –80 °C before processing. DNA was extracted from the fecal samples using a GeneJET PCR Purification Kit (key resources table) according to the manufacturer’s instructions and normalized to 2 ng/μL. To quantify *hdhA, cgh, baiA, baiB, baiCD, baiE, baiF, baiH*, and *baiI* relative to the total bacterial DCA in a sample, qPCR was performed for the 16S rRNA and *hdhA, cgh, baiA, baiB, baiCD, baiE, baiF, baiH*, and *baiI* genes using genomic DNA as templates. The sequences are shown in Supplementary Table S6.

### Statistics

GraphPad Prism version 8.0 (GraphPad Software, San Diego, CA, USA) was used for statistical analysis. Experimental values are presented as the means ± SDs. Appropriate statistical analyses were applied, assuming a normal sample distribution. When the two groups were compared, statistical significance was determined using a two-tailed Student’s *t*-test. When more than two groups were investigated, one-way analysis of variance with Tukey’s correction was applied for comparisons between different preservation groups. *P* values < 0.05 were considered significant.

## Supplementary information

Supplementary Information
