# Supplementary material for: GV-971 remodels the gut microbiota-bile acid-FXR axis to ameliorate obesity and metabolic dysfunction
Source: Cell Discov. 2026 Jul 21;12:53. doi: 10.1038/s41421-026-00904-6 (PMC13385948; doi:10.1038/s41421-026-00904-6)
Supplement: Supplementary file 1 — Supplementary Information [file 41421_2026_904_MOESM1_ESM.pdf]

Supplementary Figures and Figure legends

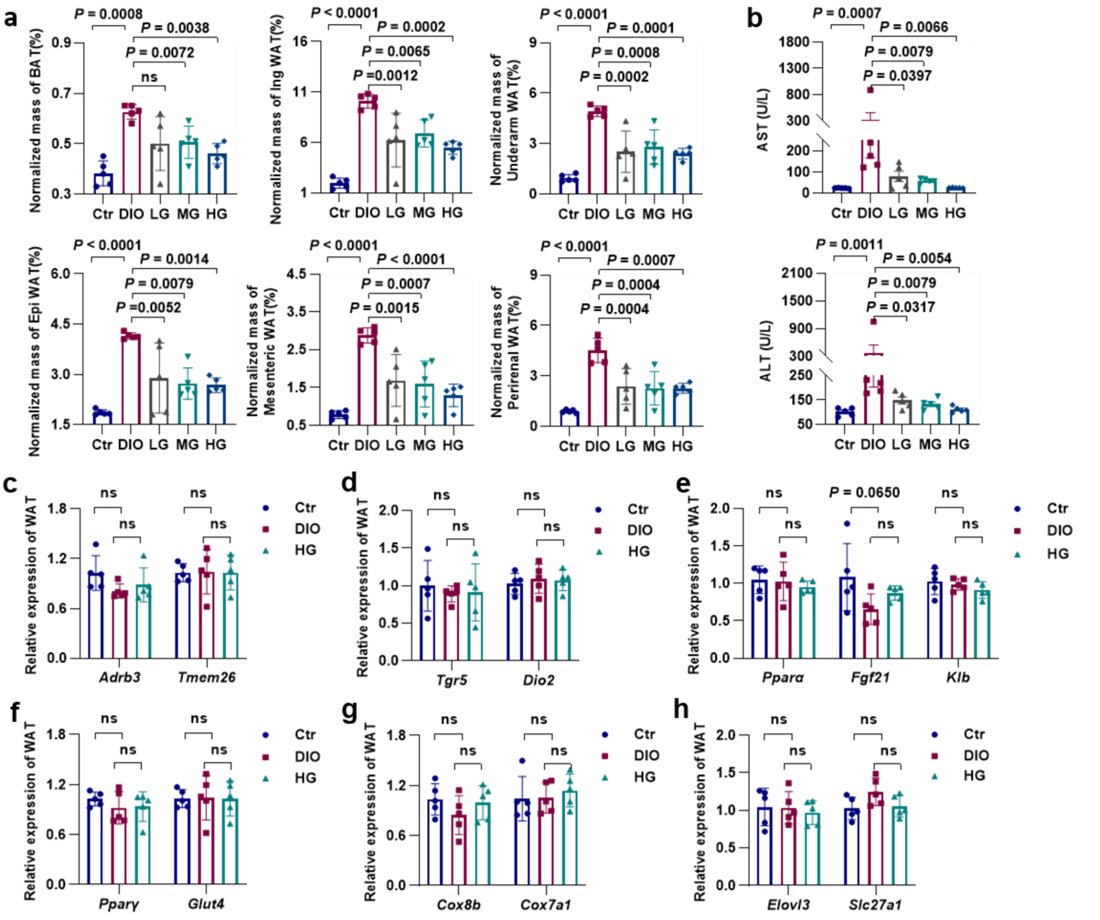

Supplementary Fig. S1. GV-971 reduces the accumulation of fat in DIO mice. (**a**) Normalized mass of Subcutaneous fat weight and Normalized mass of visceral fat weight. (**b**) Serum AST and ALT. (**c**)-(**h**) The mRNA expression of thermogenic genes in inguinal WAT of mice.

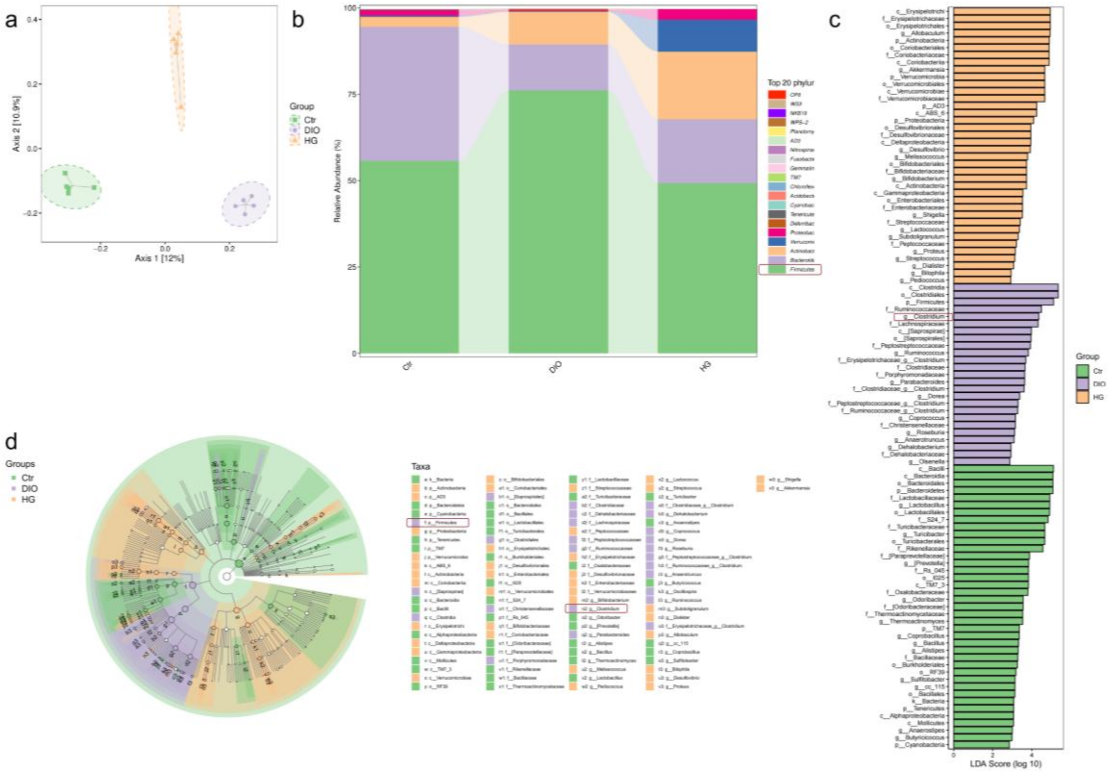

Supplementary Fig. S2. GV-971 modifies gut microbial richness and community structure in DIO mice. (**a**) Fecal samples were collected from various groups, including control mice (Ctr, n = 5), HFD-fed obese mice with vehicle (DIO, n = 5), and DIO mice treated with vehicle with a high dose of GV-971(400 mg/kg) (HG, n = 5). 16S rRNA analysis was conducted using the Illumina MiSeq platform. Principal coordinate analysis based on OTU level in different groups (n = 5). https://www.kdocs.cn/l/cnti1gCl81xJ. (**b**) Bar plots illustrating differential bacterial richness at the phylum level in different groups (n = 5). https://www.kdocs.cn/l/ci8VeX2042up. (**c**) Bar chart of LDA effect values for the indicator species in different groups (n = 5). The vertical axis shows the classification units with significant differences between groups, while the horizontal axis presents the log scores from the LDA analysis for each classification unit in a bar chart for a more intuitive display. https://www.kdocs.cn/l/cqJW7CSCA0oY. (**d**) Display a chart of inter-

group differential classification units based on the classification hierarchy tree. https://www.kdocs.cn/l/ceCHIgKHDEpZ.

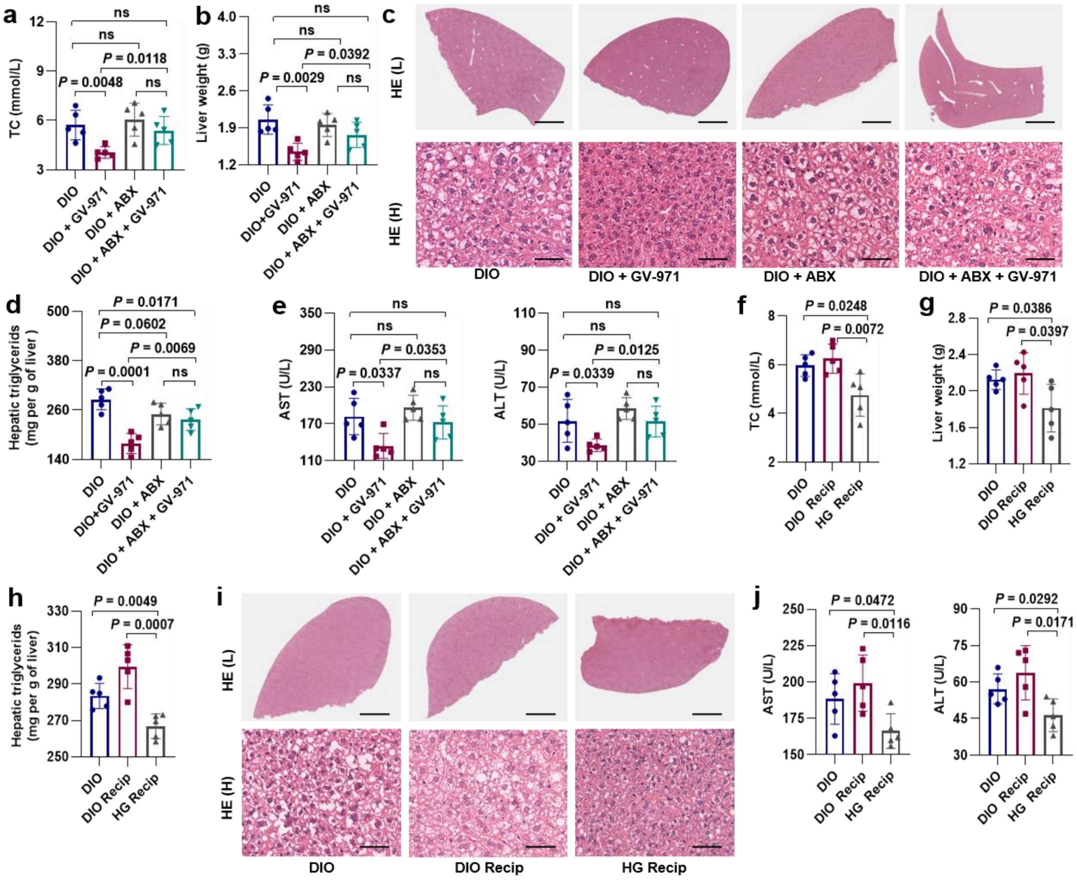

Supplementary Fig. S3. Clearing the intestinal microbiota eliminates the effect of GV-971 in improving the metabolic function of DIO mice (a-e). (**a**) Serum total cholesterol level. (**b**) Liver weight. (**c**) Representative H&E staining of liver sections. Scale bars: 100 μm (HE(L): 2000μm). (**d**) Liver triglyceride contents. (**e**) Serum AST and ALT. **Transplantation of gut microbiota regulated by GV-971 improves metabolic dysfunction in DIO mice (f-j).** (**f**) Serum total cholesterol level. (**g**) Liver weight. (**h**) Liver triglyceride contents. (**i**) Representative H&E staining of liver sections. Scale bars: 100 μm (HE(L): 2000μm). (**j**) Serum AST and ALT.

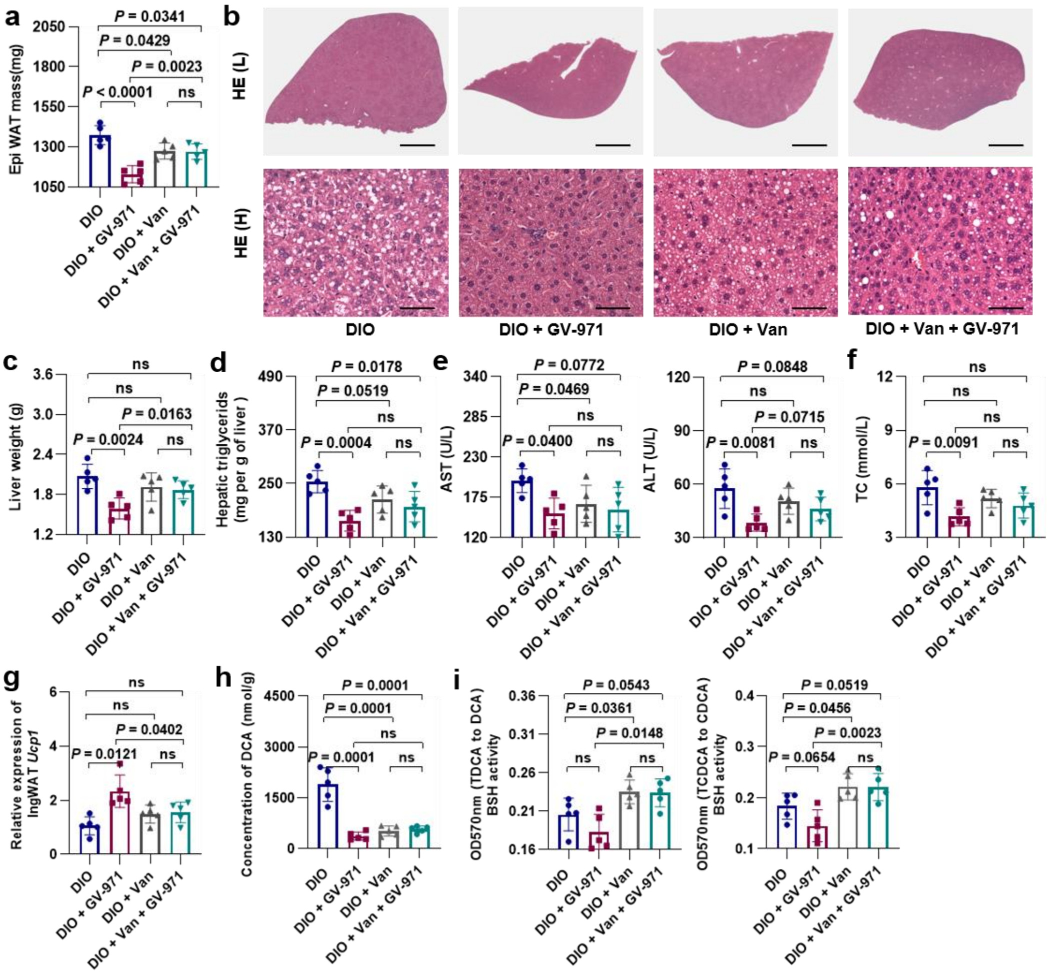

Supplementary Fig. S4. The effect of GV-971 in improving metabolic dysfunction is significantly weakened in DIO mice lacking *Clostridium*. (**a**) Mass of the epididymis WAT. (**b**) Representative H&E staining of liver sections. Scale bars: 100 μm (HE(L): 2000μm). (**c**) Liver weight. (**d**) Liver triglyceride contents. (**e**) Serum AST and ALT. (**f**) Serum total cholesterol level. (**g**) mRNA expression of *Ucp1* thermogenic genes in inguinal WAT. (**h**) The intestinal DCA levels (nmol/g). (**i**) Gut microbial BSH activity analyzed by ninhydrin assay (TDCA to DCA) (left). Gut microbial BSH activity analyzed by ninhydrin assay (TCDCA to CDCA) (right).

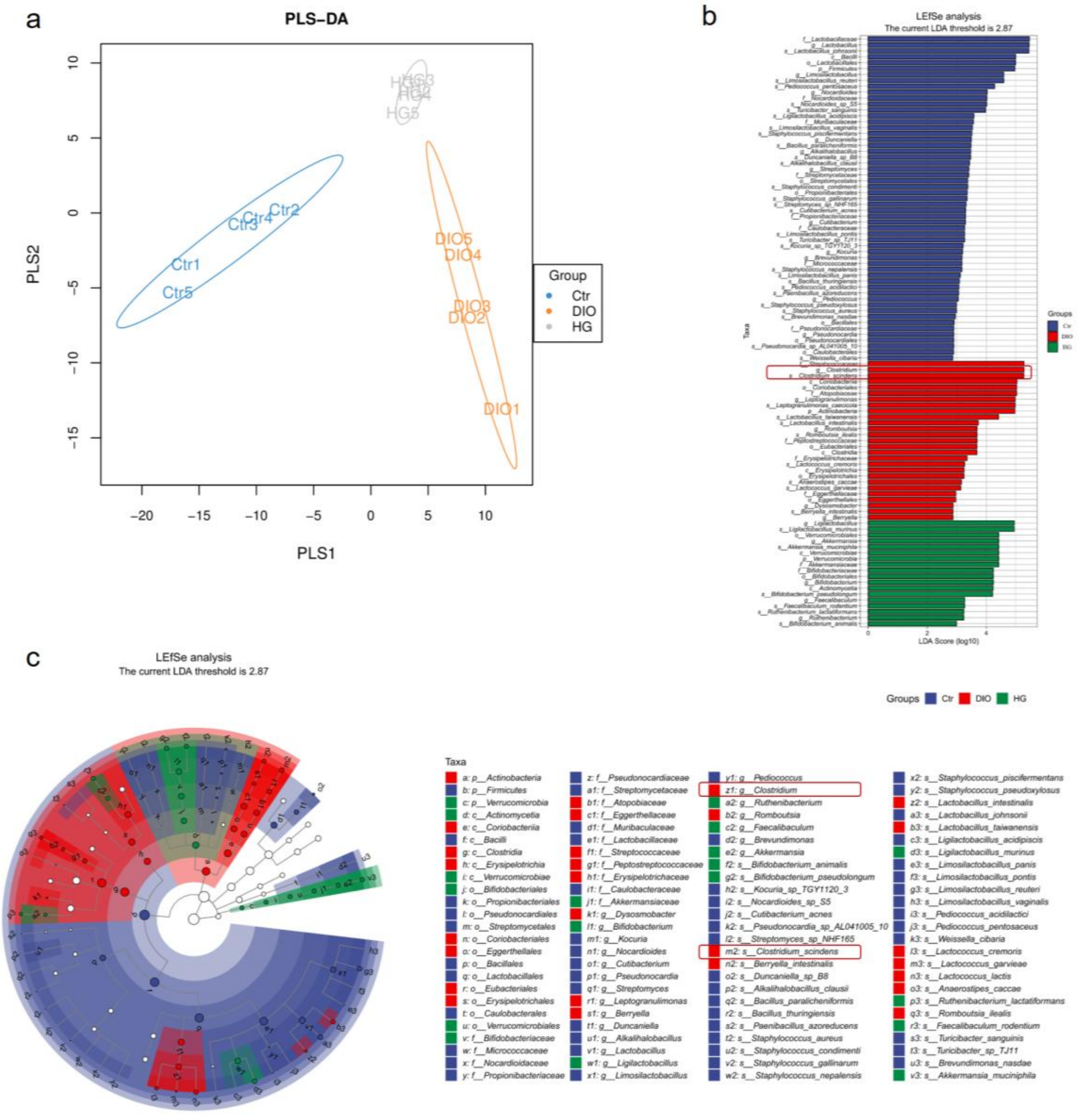

Supplementary Fig. S5. GV-971-induced downregulation of *Clostridium scindens* abundance in DIO mice. (**a**) Fecal samples were collected from various groups, including control mice (Ctr, n = 5), HFD-fed obese mice with vehicle (DIO, n = 5), and DIO mice treated with vehicle with a high dose of GV-971(400 mg/kg) (HG, n = 5). Metagenomic analysis was conducted using the Illumina MiSeq platform. Partial least-square discriminant analysis (PLS-DA). (**b**) Bar chart of LDA effect values for the indicator species in different groups. The vertical axis represents the classification units that have significant differences between groups, while the horizontal axis presents the log scores of the LDA analysis for each classification unit in a bar chart form for a more

intuitive display. (**c**) Display chart of inter-group differential classification units based on classification hierarchy tree.

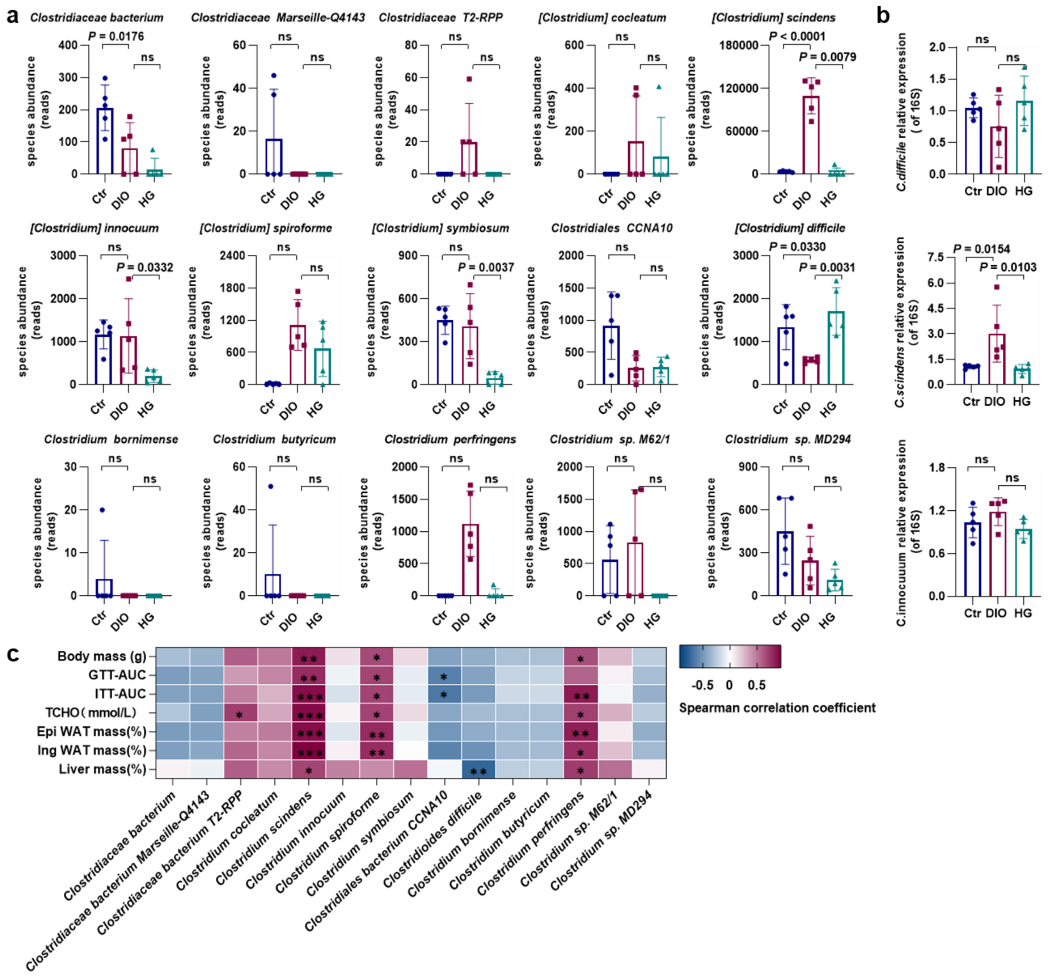

Supplementary Fig. S6. GV-971-induced downregulation of *Clostridium scindens* abundance is negatively correlated with obesity and metabolic dysfunction in DIO mice. (**a**). Different *Clostridium* species abundance (reads) of *Clostridium* based on metagenomics data. (**b**). The relative expression level of the species in relation to 16S by qPCR. (**c**) A Spearman correlation heatmap demonstrating the correlation between *Clostridium* species and obesity-related indicators, with positive correlations in red and negative correlations in blue. Data are presented as mean ± SD. Statistical comparisons were performed using one-way ANOVA with Dunnett's test for statistical significance.

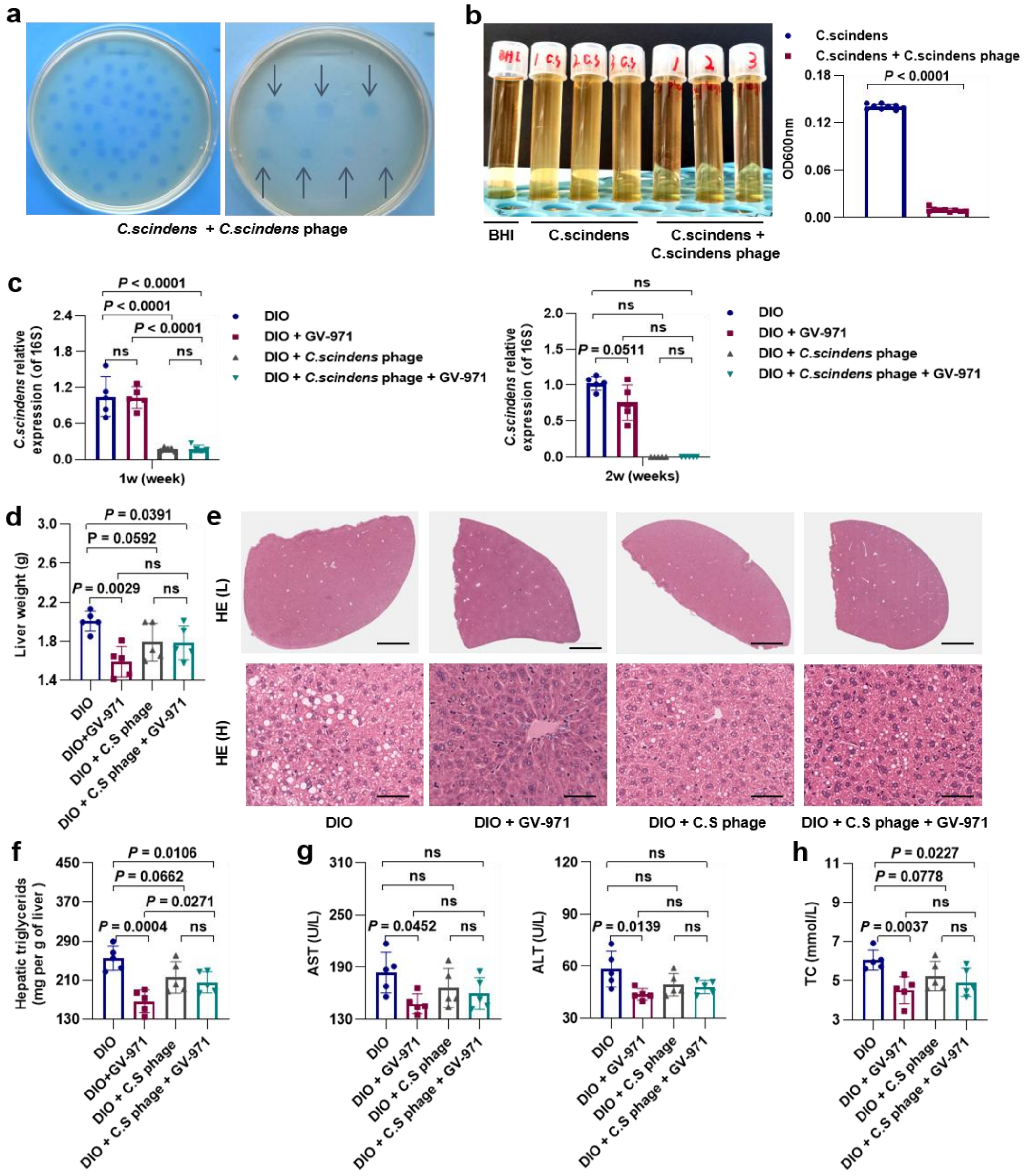

Supplementary Fig. S7. The effect of GV-971 in improving metabolic dysfunction is significantly weakened in DIO mice with *C.scindens* phage implanted. (a) Bacteriophage plaque: a culture of *C. scindens* grown overnight was mixed with 4 mL of BHI top agar (0.4% agar) and poured onto the surface of a BHI agar plate (1.5% agar). The phage was co-cultured with *C. scindens* (left). The phage was diluted and spotted on the plate (right). After overnight growth at 37 °C, images were captured. **(b)** Turbidity: The phage was co-cultured with C. scindens in BHI after overnight growth

at 37 °C. The appearance of bacterial turbidity (left). The OD600nm of BHI (right). (**c**) The relative expression level of *C. scindens* in relation to 16S by qPCR after *C. scindens phage* colonization. (**d**) Liver weight. (**e**) Representative H&E staining of liver sections. Scale bars: 100 μm (HE(L): 2000μm). (**f**) Liver triglyceride contents. (**g**) Serum AST and ALT. (**h**) Serum total cholesterol level.

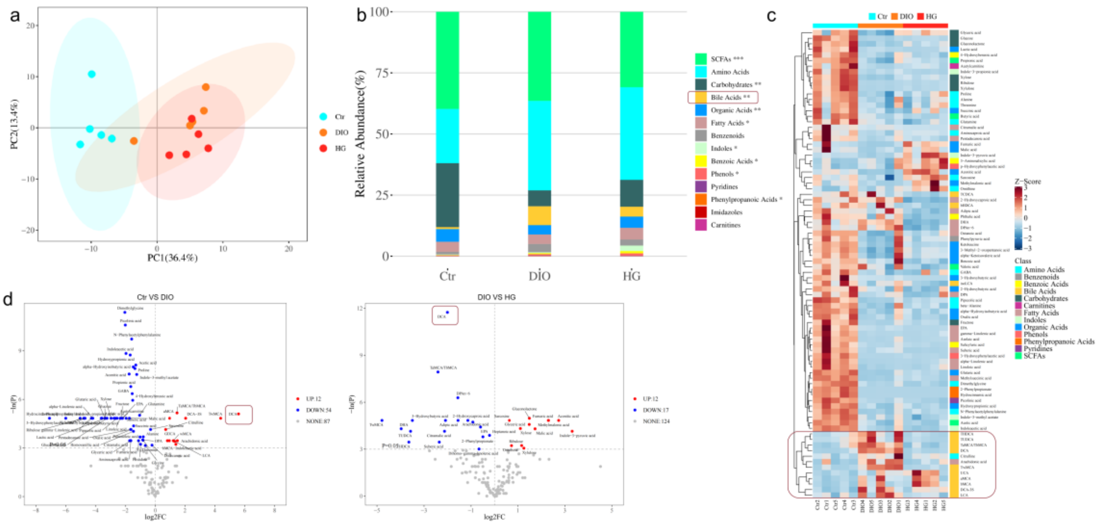

Supplementary Fig. S8. GV-971 reduces the concentration of DCA in the intestines of DIO mice. (**a**) Fecal samples were collected from various groups, including control mice (Ctr, n = 5), HFD-fed obese mice with vehicle (DIO, n = 5), and DIO mice treated with vehicle with a high dose of GV-971(400 mg/kg) (HG, n = 5). Analysis was conducted using UPLC-MS/MS. Results of a partial least squares discriminant analysis model based on the metabolic profiles of fecal samples in different groups (n = 5). (**b**) A percentage chart illustrating the relative abundance of metabolite classes in various groups. (**c**) A heatmap showing potential differential metabolites in various groups (n = 5). https://www.kdocs.cn/l/ck6axufmpltf. (**d**) Volcano plots depicting 153 metabolites between the Ctr group and DIO group (left). https://www.kdocs.cn/l/cbVct0rC43lw, and between the DIO group and HG group (right). https://www.kdocs.cn/l/cjEwjUMLqFEG.

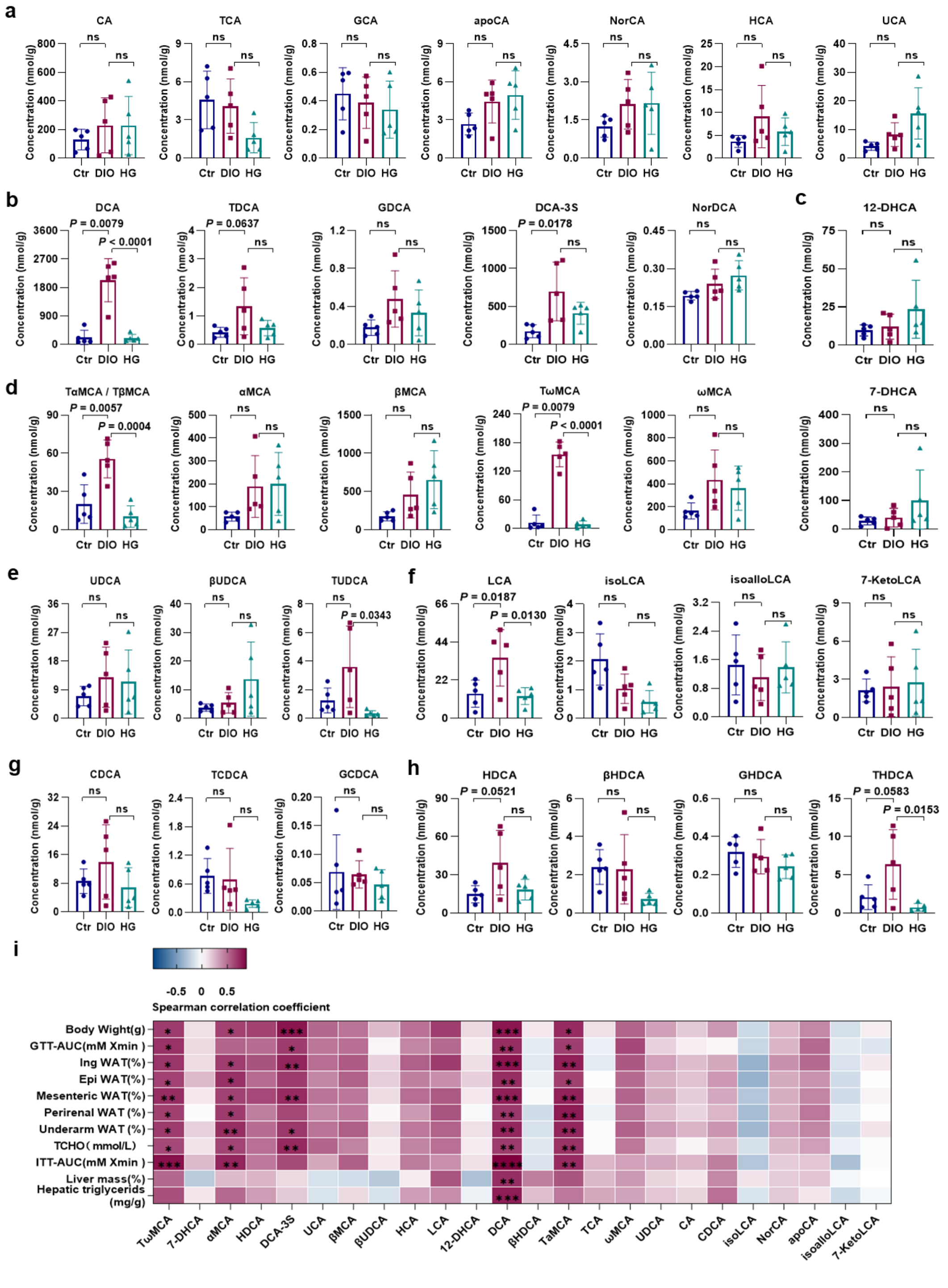

Supplementary Fig. S9. GV-971 modulates the bile acid profile of DIO mice. (a-h) Bile acid levels. (**i**) A Spearman correlation heatmap demonstrating the correlation between Bile acid and obesity-related indicators, with positive correlations in red and negative correlations in blue. Data are presented as mean ± SD. Statistical comparisons were performed using one-way ANOVA with Dunnett's test for statistical

significance.

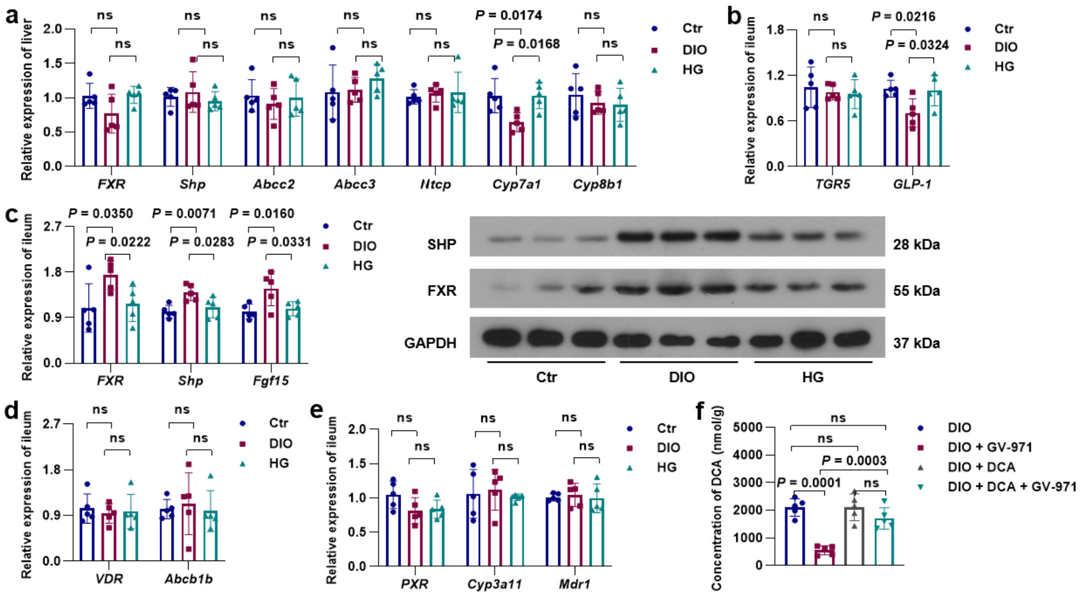

Supplementary Fig. S10. The effects of GV-971 on the bile acid signaling pathways in the liver and intestines of DIO mice. (**a**) The mRNA expression levels of bile acid signaling molecules in the liver. (**b**)The mRNA expression levels of bile acid signaling molecules in the ileum. (**c**) The mRNA expression levels of bile acid signaling molecules in the ileum (left) and Western blot analysis (cropping of blot images) of FXR and SHP protein expression in intestinal (right) of different groups, including control mice treated with vehicle (Ctr, n = 5), DIO mice treated with vehicle (DIO, n = 5), and DIO mice treated with GV-971(400 mg/kg) (HG, n = 5). (**d,e**) The mRNA expression levels of bile acid signaling molecules in the ileum. (**f**) The intestinal DCA levels (nmol/g).

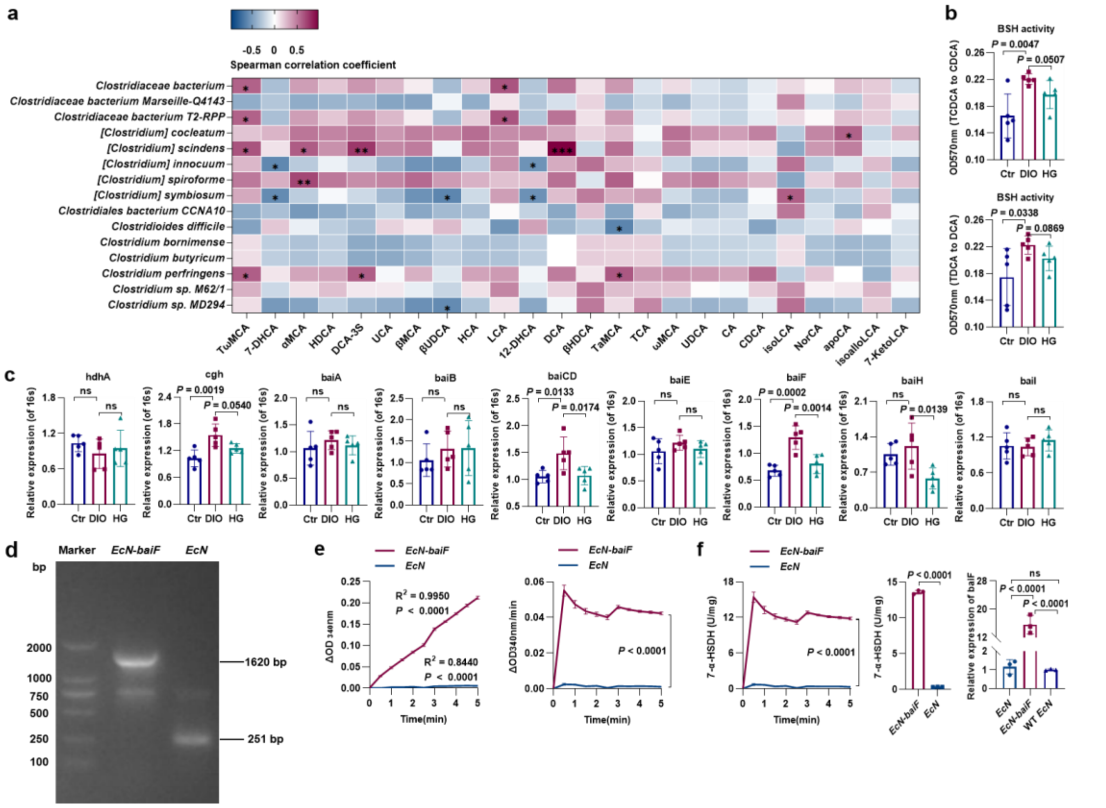

Supplementary Fig. S11. GV-971-induced down regulation of *Clostridium scindens* abundance is positive correlated with GV-971-modulated bile acid profile (a-c). (**a**) A Spearman correlation heatmap demonstrating the correlation between *Clostridium* species and Bile acid, with positive correlations in red and negative correlations in blue. Data are presented as mean ±SD. Statistical comparisons were performed using one-way ANOVA with Dunnett's test for statistical significance. (**b**) Gut microbial BSH activity analyzed by ninhydrin assay (TCDCA to CDCA) (up), Gut microbial BSH activity analyzed by ninhydrin assay (TDCA to DCA) (down). (**c**) Relative expression level of bile acid conversion genes. **The engineered *EcN-baiF* overexpresses the *baiF* gene and effectively produces 7-α HSDH (d-f).** (**d**) The product identification results of *EcN-baiF*. (**e**) The OD value of the reaction system, when using the direct colorimetric method to detect the enzymes activities of 7-α HSDH in the bacterial

culture solution of *EcN-baiF*. (**f**) The enzymes activities of 7-α HSDH.

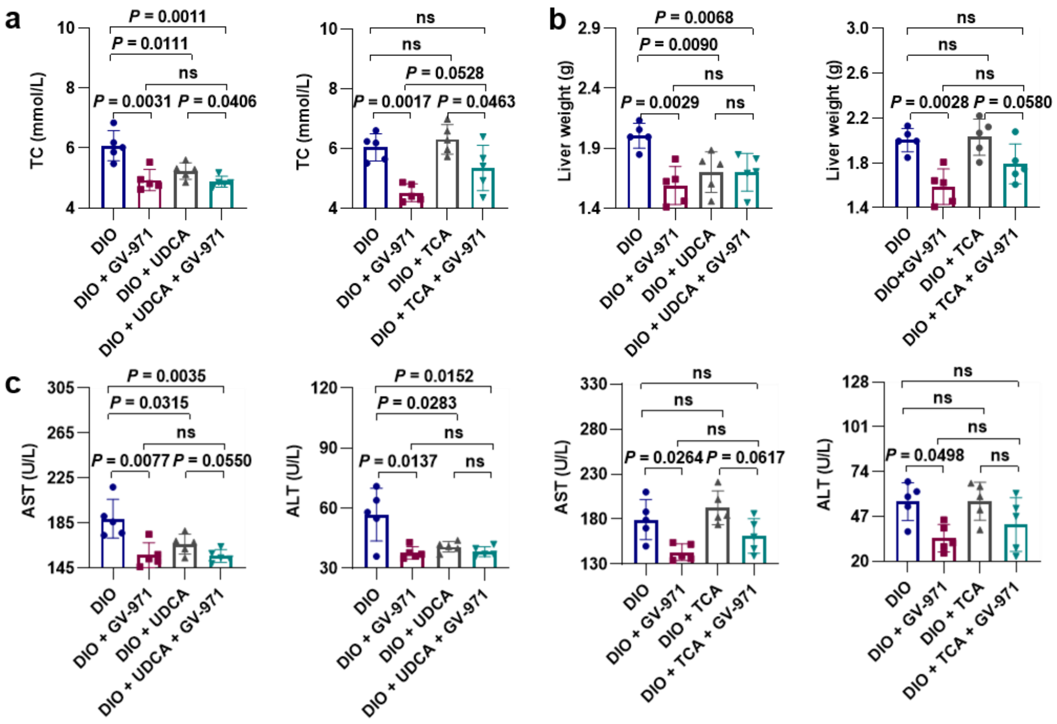

Supplementary Fig. S12. The effects of GV-971 on the metabolism of serum and liver in DIO mice, when the FXR signaling molecules in the intestines of DIO mice are inhibited or activated. (**a**) Serum total cholesterol level. (**b**) Liver weight. (**c**) Serum AST and ALT.

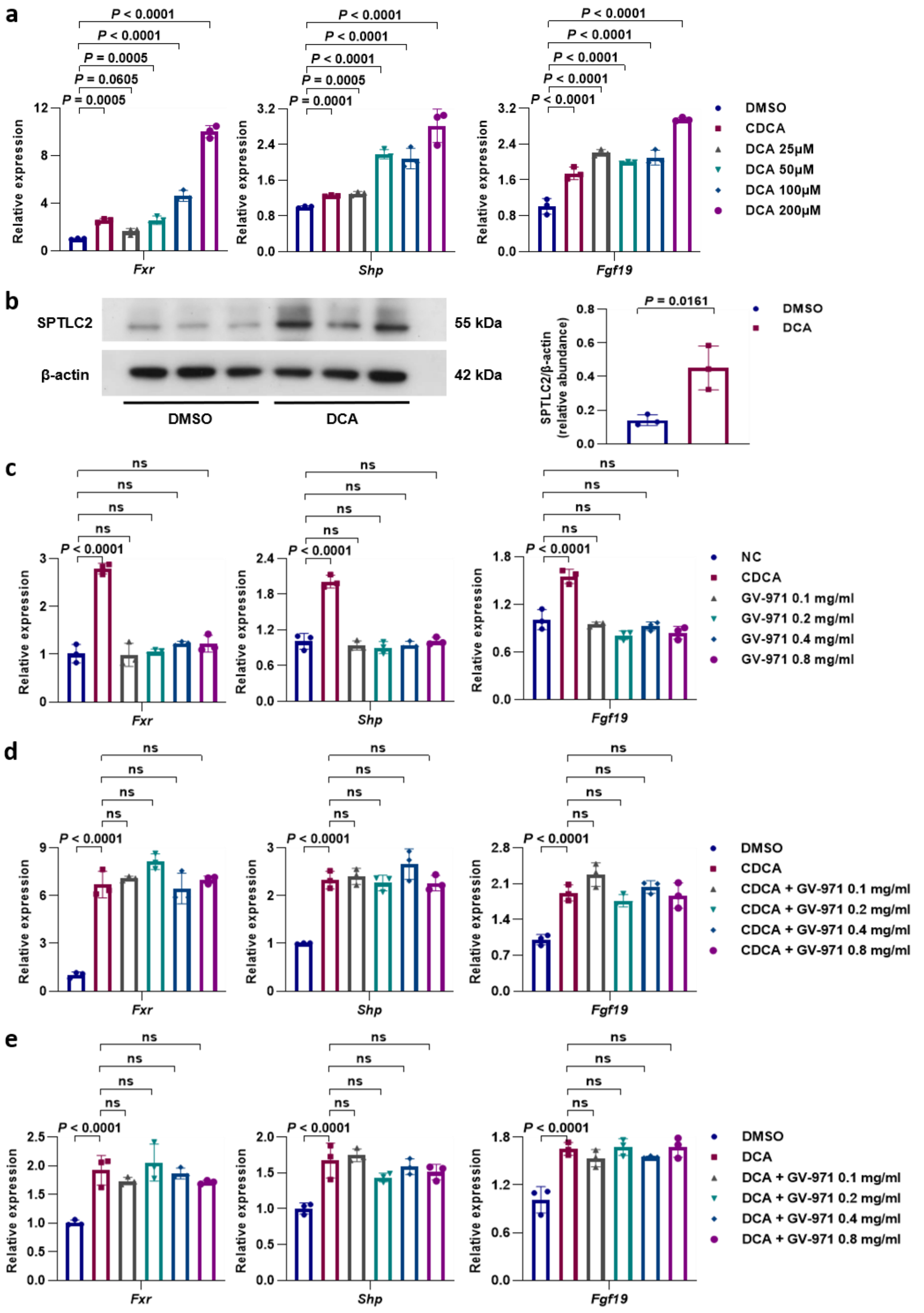

Supplementary Fig. S13. The effect of DCA on FXR in intestinal epithelial cell. (a) *Shp*, *Fgf19* and *Fxr* mRNA expression in differentiated Caco-2 cells after treatment with different concentrations of DCA and Fxr agonist CDCA (20 μM). n = 3

replicates/treatment. (**b**) Western blot analysis of SPTLC2 protein expression in Caco-2 cells after treatment with DMSO and DCA (50 μM) and quantitation of SPTLC2 expression. (**c**) *Shp*, *Fgf19* and *Fxr* mRNA expression in differentiated Caco-2 cells after treatment with different concentrations of GV-971. n = 3 replicates/treatment. (**d**) *Shp*, *Fgf19*, and *Fxr* mRNA expression in differentiated Caco-2 cells after treatment with different concentrations of GV-971with Fxr agonist CDCA (20 μM) treatment. n = 3 replicates/treatment. (**e**) *Shp*, *Fgf19*, and *Fxr* mRNA expression in differentiated Caco-2 cells after treatment with different concentrations of GV-971with DCA (50 μM) treatment. n = 3 replicates/treatment.

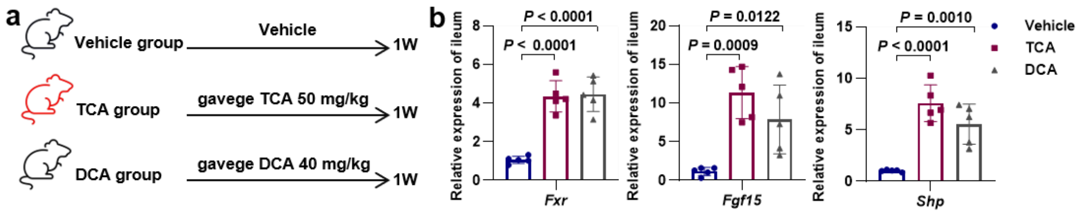

Supplementary Fig. S14. The effect of DCA on the Fxr in the intestinal of C57 mice.

(**a**) Schematic experimental procedure: C57 mice were classified into three groups, including C57 mice treated with vehicle (Vehicle, n = 5), C57 mice treated with TCA (n = 5) for 1 week, and C57 mice treated with DCA (n = 5) for 1 week. (**b**) *Shp*, *Fgf19*, and *Fxr* mRNA expression in the intestine of C57 mice after treatment with vehicle, TCA (50 mg/kg), and DCA (40 mg/kg).

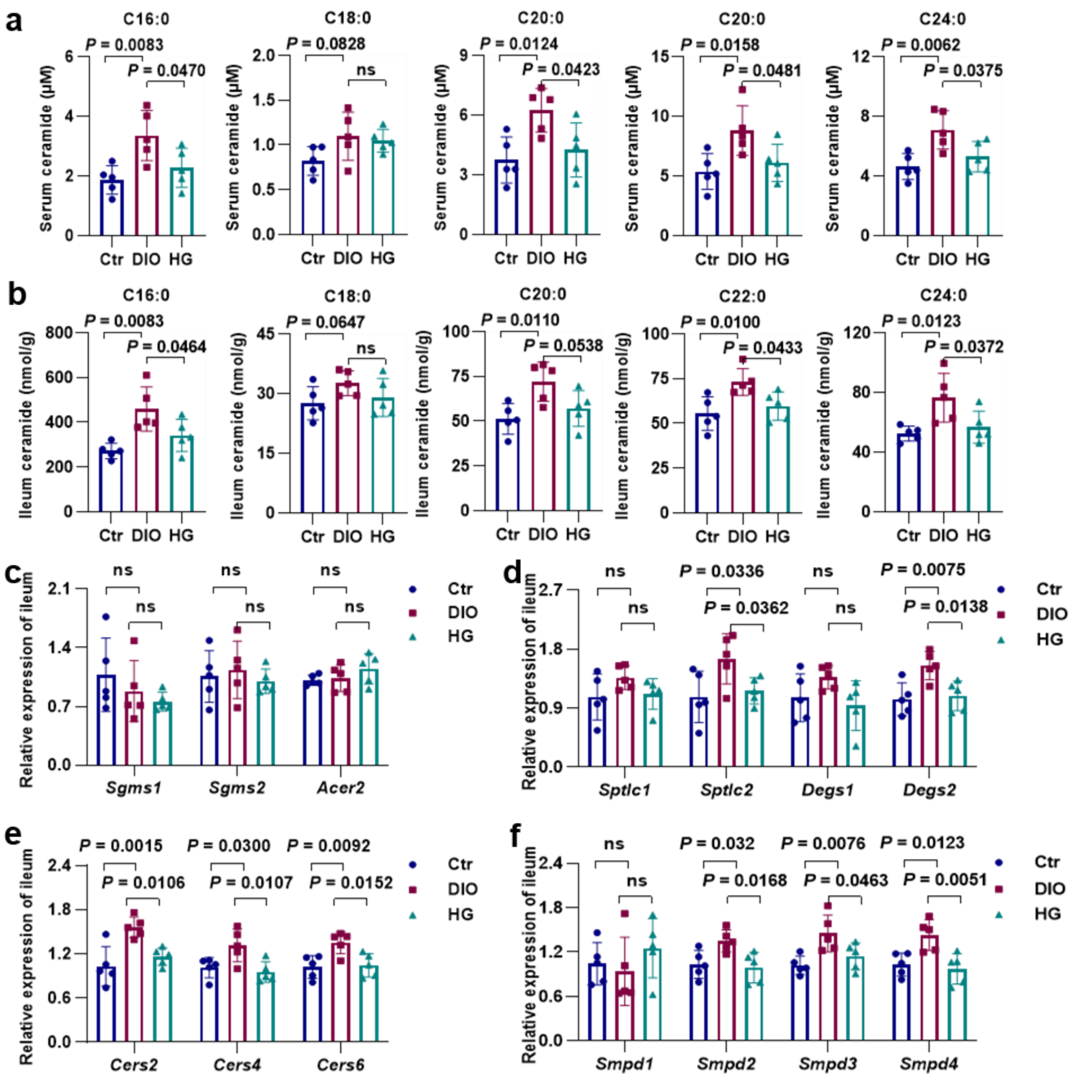

Supplementary Fig. S15. The effects of GV-971 on ceramides. (**a**) The level of ceramides in the serum. (**b**) The level of ceramides in the ileum. (**c-f**) The mRNA expression of genes involved in the synthesis and decomposition of intestinal ceramides, which are downstream targets of the FXR signaling molecule in the bile acid pathway of DIO mice.

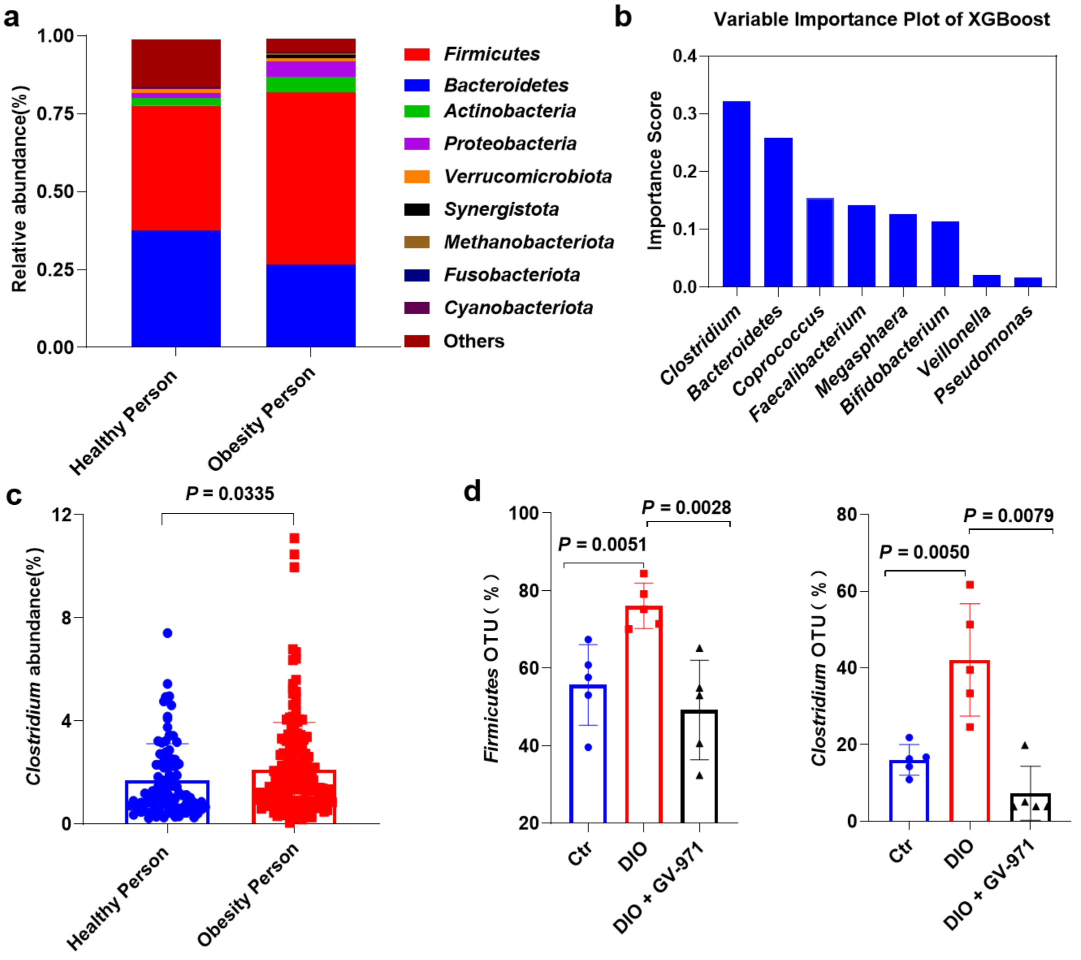

Supplementary Fig. S16. The abundance of Clostridium in obese patients is significantly higher than that in healthy individuals. Data are collected and analyzed from metagenomic cohort data from public databases (Gmrepo, https://gmrepo.humangut.info/). Data for obese patients (n = 167) and healthy individuals (n = 95) were from the project: PRJEB4336. (**a**) Bar plots illustrating differential bacterial richness at the phylum level between obese patients and healthy individuals. (**b**) Variable Importance Plot of XGBoost, which uses the XGBoost model to calculate the importance of bacteria. (**c**) Histogram depicting the disparity in Clostridium abundance between obese patients and healthy individuals. (**d**) Histogram depicting the disparity in Clostridium abundance between DIO mice and DIO mice

treated with GV-971.

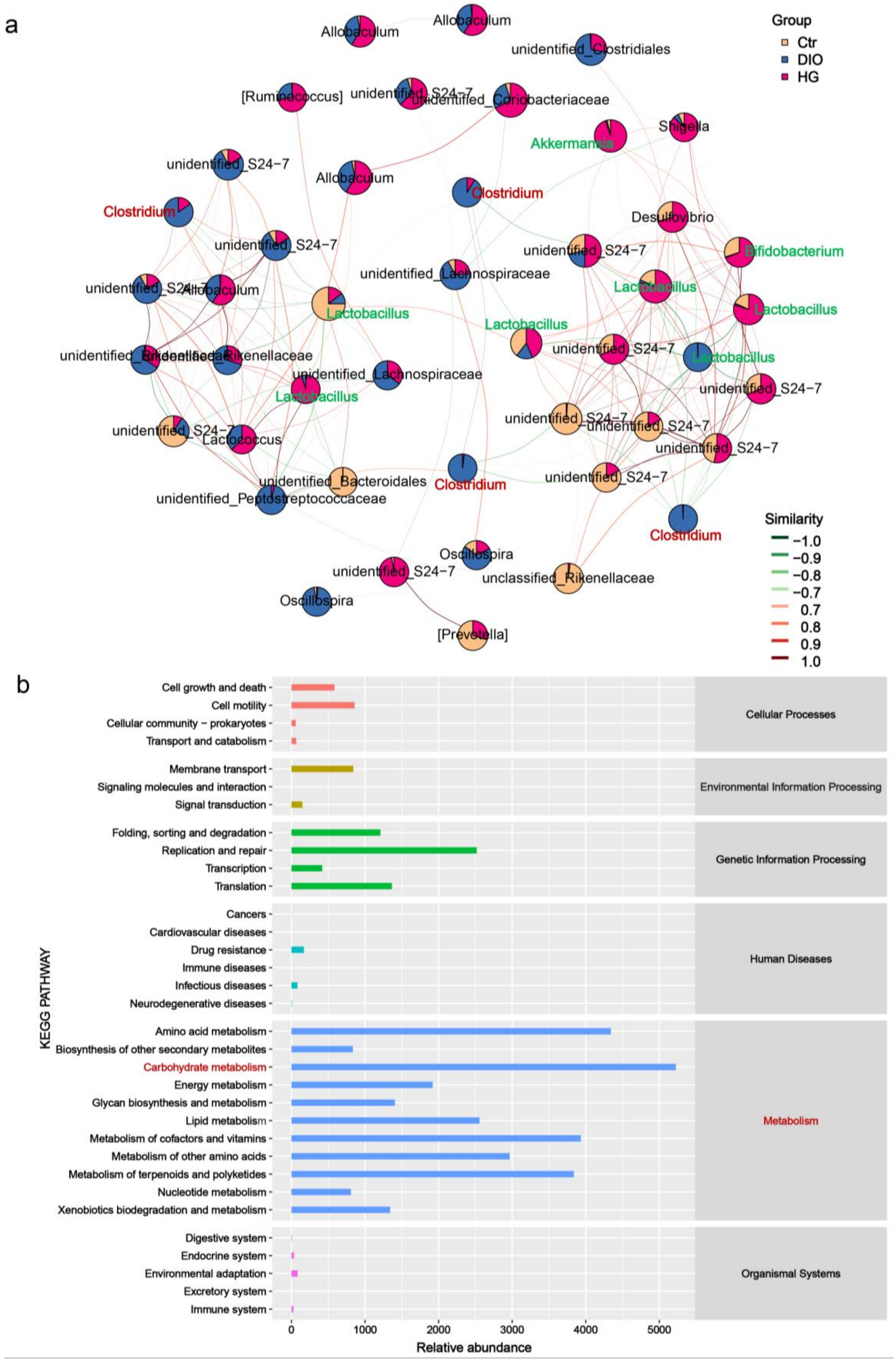

Supplementary Fig. S17. Analysis of the association network of microbial dominant species and prediction of 16S rRNA gene sequences in the functional

database. (**a**) The advantage of the grouped abundance pie chart is the phylogenetic network diagram. Nodes represent ASV or OTU in the samples. The size of the nodes is proportional to their abundance (measured in log2(CPM/n)). In the figure, only the top 50 ASVs/OTUs with the highest average abundance in the sample are shown, presented in the form of a pie chart to display the relative abundance ratio of this node in different groups. The connections between nodes indicate the correlation between the two connected nodes. Red lines indicate positive correlation, while green lines indicate negative correlation. (**b**) Predicted abundance map of KEGG secondary functional pathways. The horizontal axis represents the abundance of the functional pathways (in units of per million KO), the vertical axis represents the functional pathways at the second level of KEGG classification, and the rightmost column represents the first-level pathway to which this pathway belongs.

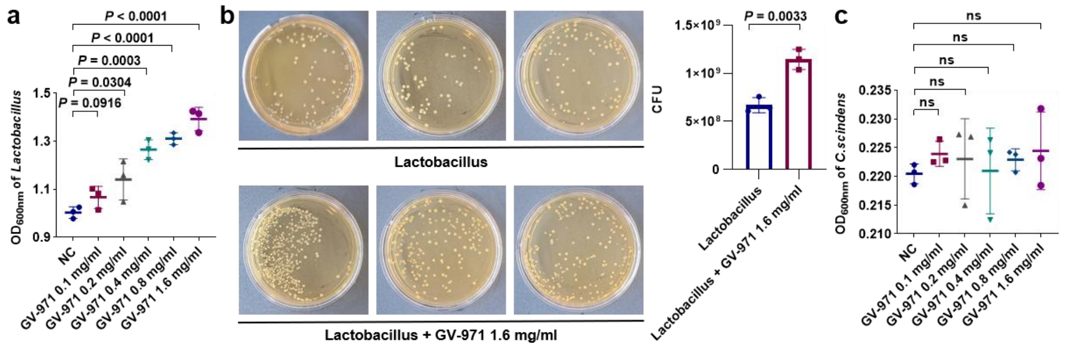

Supplementary Fig. S18. The effect of GV-971 on *Lactobacillus* and *Clostridium*.

(**a**) Turbidity: OD600nm of *Lactobacillus* cultured with different concentrations of GV-971. (**b**) Lactobacillus colony (left) and CFU (right) cultured with or without GV-971. (**c**) Turbidity: OD600nm of *Clostridium* cultured with different concentrations of GV-971.

Supplementary Table S1. Longitudinal dose-response analysis of body weight by Linear Mixed-Effects (LME) modeling

Effect	Estimate (SE)	df	t value	p value
Intercept	23.27 (1.83)	29.6	12.74	<0.001
dose	-0.0720 (0.00655)	29.6	-10.99	<0.001
week	1.251 (0.0761)	279.5	16.44	<0.001
dose:week	-0.00507 (0.00027)	279.5	-18.78	<0.001

Supplementary Table S2. Effect size comparison by Cohen’s d

Group	Cohen’s d	95%CI	Effect size
LG vs HG	0.942	-3.975 to 14.53	Large
MG vs HG	0.809	-5.655 to 12.85	Large
LG vs MG	-0.269	-7.575 to 10.93	Small

Supplementary Table S3. The weight-loss effect for each dose

Dose (mg/kg)	Mean / median Body Weight (g)	Weight Loss Effect (g)
0	53.54 / 53.6	0 / 0
100	43.58 / 44.4	9.96 / 9.2
200	41.90 / 40.2	11.64 / 13.4
400	38.30 / 39.6	15.24 / 14.0

Supplementary Table S4. Plateau effect analysis by Emax Model Fitting (Hill coefficient γ = 1)

$Emax$ (g)	$ED50$ (mg/kg)	R^2	$ED95$ (mg/kg)	$E(400)$ (g)
16.2	68.5	0.98	1301.5	13.8

Supplementary Table S5. Weekly weight-loss effects (DIO - HG mean)

Week	Effect (g)	Weekly increment (g)
11	1.00	–
12	3.14	+2.14
13	3.84	+0.70
14	4.54	+0.70
15	6.96	+2.42
16	7.68	+0.72
17	8.54	+0.86
18	8.24	– 0.30
19	8.64	+0.40
20	9.44	+0.80
21	13.02	+3.58
22	13.72	+0.70
23	15.92	+2.20
24	16.34	+0.42
25	15.24	– 1.10

Supplementary Table S6. Sequences of the qPCR primers used in this study

Primers	Forward	Reward
**Mouse primers**		
Ucp1	GCCTCTACGACTCAGTCCAA	CATTAGATTAGGGGTCGTCC
Pgc1a	TATGGAGTGACATAGAGTGTGCT	CCACTTCAATCCACCCAGAAAG
Prdm16	CCACCAGCGAGGACTTCAC	GGAGGACTCTCGTAGCTCGAA
Adrb3	GGCCCTCTCTAGTTCCCAG	TAGCCATCAAACCTGTTGAGC
Tmem26	ATGGTGCATTTCAAGAAGCC	GCTCACCCTCAAGTTCAAGC
Dio2	AATTATGCCTCGGAGAAGACCG	GGCAGTTGCCTAGTGAAAGGT
Tgr5	CCTGGCAAGCCTCATCGTC	AGCAGCCCGGCTAGTAGTAG
CD40	TTGTTGACAGCGGTCCATCTA	CCATCGTGGAGGTACTGTTTG
CD147	CGTGCAGAACTCCTGTGATAAC	GTCCACCTATGCTGGAGAAGG
PPARα	ACAACGCGATTCGTTTTGGA	GGCCAGAGATTTGAGATCTGCA
Fgf21	CTGCTGGGGGTCTACCAAG	CTGCGCCTACCACTGTTCC
Klb	TGTTCTGCTGCGAGCTGTTAC	CCGGACTCACGTACTGTTTTT
Pparγ	AGCCTCATGAAGAGCCTTCCA	ACCCTTGCATCCTTCACAAGC
Glut4	GTGACTGGAACACTGGTCCTA	CCAGCCACGTTGCATTGTAG
Cox8b	GAACCATGAAGCCAACGACT	GCGAAGTTCACAGTGGTTCC
Cox7a1	CAGCGTCATGGTCAGTCTGT	AGAAAACCGTGTGGCAGAGA
Elovl3	TTCTCACGCGGGTTAAAAATGG	GAGCAACAGATAGACGACCAC
Slc27a1	CTGGGACTTCCGTGGACCT	TCTTGCAGACGATACGCAGAA
Fxr	TGGGCTCCGAATCCTCTTAGA	TGGTCCTCAAATAAGATCCTTGG

Shp	TCTGCAGGTCGTCCGACTATTC	AGGCAGTGGCTGTGAGATGC
Fgf15	GCCATCAAGGACGTCAGCA	AGGCAGTGGCTGTGAGATGC
PXR	TAGGGACCTGCCTATTGAGGA	CCGTTTCCGTGTCGAACATC
Cyp3a11	CATTCGCCCTGGTACTTCTC	TCTCCATTTCCACGGTCAGT
Mdr1	GGTGGTGTCATTGTGGAGCAAG	GCATCAGTGTCACTCTGGGATC
Vdr	CACAGTTCGCCTAGCTTCTC	GAGGACAGGACACCATCATC
Abcb1b	GATGGCTACAGCTTGAAGACCT	CAATCACCACCACATCAAGCC
GLP-1	GCACACAGTGCCCTAACCT	TTTAGCTGCTTCCGTGGTCTT
Abcc2	ACGTTTAGTTGGTATGACAGCAC	TGCTTCTTGGTCAATCCGTGT
Abcc3	GTCCCCTGCATCTACCTGTG	GCCGTCTTGAGCCTGGATAA
Ntcp	CAAACCTCAGAAGGACCAAACA	GTAGGAGGATTATTCCCGTTGTG
Cyp7a1	AACAACCTGCCAGTACTAGATAGC	GTGTAGAGTGAAGTCCTCCTTAGC
Cyp8b1	CTAGGGCCTAAAGGTTCGAGT	GTAGCCGAATAAGCTCAGGAAG
Sptlc1	CGAGGGTTCTATGGCACATT	GGTGGAGAAGCCATACGAGT
Sptlc2	TCACCTCCATGAAGTGCATC	CAGGCGTCTCCTGAAATACC
Degs1	GACGGCTACTGGGATCTGA	TCACCACACACCAATTCAGG
Degs2	CGCAGCGAAAACAAGAATAA	GAAGATGTCTTGGAGGGCTG
Cers2	AAGTGGGAAACGGAGTAGCG	ACAGGCAGCCATAGTCGTTC
Cers4	GGATTAGCTGATCTCCGCAC	CCAGTATGTCTCCTGCCACA
Cers6	AAGCCAATGGACCACAAACT	TGCTTGGAGAGCCCTTCTAAT
Smpd1	GTTACCAGCTGATGCCCTTC	AGCAGGATCTGTGGAGTTG
Smpd2	AGCAGGATCTGTGGAGTTG	CTCCAGCCATGAAGCTCAAC

Smpd3	CCTGACCAGTGCCATTCTTT	AGAAACCCGGTCCTCGTACT
Smpd4	ACCTGGCCCTCAATCCATTTG	ATAGGCACAGTCCGAAGTACG
Gapdh	CATCACTGCCACCCAGAAGACTG	ATGCCAGTGAGCTTCCCGTTCAG
**Bacterial primers**		
16S	AGAGTTTGATCCTGGCTCAG	AAGTCGTAACAAGGTAACC
C. scinden	GCAACCTGCCTTGCACT	ACCGAATGGCCTTGCCA
hdhA	ATGAAAAAAATTATTAGTA	TTATTTCTCTTCCTTCTCACC
cgh	ATGAAAAAGTAGTAGGAAAAAC	TTATTCCTCTACTTTTCCACTT
baiA	GGCTGAAGGTATTGCTGGTG	CGTAGCCATCCACTTCACCT
baiB	GGAAGGTTTGCTGGGATGGT	CCGATACCGGACCTTGAAGA
baiCD	CAGATGGTATTCCGGGTGGA	TCCACATCCTCCACCTTCAC
baiE	ATGACCATGGCTTATGTCCG	TCAGTACCTAGCCATCCGCA
baiF	TTCAGYTTCTACACCTG	GGTTRTCCATRCCGAACAGCG
baiH	GGGACAGGAATATTGGCGCT	AACCAGCACCTCCTTCACCT
baiI	ATGAAAAAGTTACTCAAGTTC	TTACCATACCTTTTACTTGC
C.spiroform	GAAGATACCTCACCATACAC	ATCTTAGGAATACCGTCACT
C.difficile	AAAGAAGCTACTAAGGGTACAAA	CATAATATTGGGTCTATTCCTAC
C.perfrigen	TAACCTGCCTCATAGAGT	TTTCACATCCCACTTAATC
C.innocuum	AGAGTTTGATCMTGGCTCAG	GGTTACCTTGTTAACGACTT
**Human primers**		
18S	GATATGCTCATGTGGTGTTG	AATCTTCTTCAGTCGCTCCA

Shp	CCCCAAGGAATATGCCTGCC	TAGGGCGAAAGAAGAGGTCCC
Fgf19	CCAGAAGACAGGCAGTAGT	CTGGAGGGA TTTGGGAAGG
Fxr	AACCATACTCGCAATACAGCAA	ACAGCTCATCCCCTTTGATCC
